# A Functional Requirement for Astroglia in Promoting Blood Vessel Development in the Early Postnatal Brain

**DOI:** 10.1371/journal.pone.0048001

**Published:** 2012-10-24

**Authors:** Shang Ma, Hyo Jun Kwon, Zhen Huang

**Affiliations:** 1 Departments of Neurology and Neuroscience, University of Wisconsin-Madison, Madison, Wisconsin, United States of America; 2 Graduate Program in Cellular and Molecular Biology, University of Wisconsin-Madison, Madison, Wisconsin, United States of America; 3 Neuroscience Training Program, University of Wisconsin-Madison, Madison, Wisconsin, United States of America; Hôpital Robert Debré, France

## Abstract

Astroglia are a major cell type in the brain and play a key role in many aspects of brain development and function. In the adult brain, astrocytes are known to intimately ensheath blood vessels and actively coordinate local neural activity and blood flow. During development of the neural retina, blood vessel growth follows a meshwork of astrocytic processes. Several genes have also been implicated in retinal astrocytes for regulating vessel development. This suggests a role of astrocytes in promoting angiogenesis throughout the central nervous system. To determine the roles that astrocytes may play during brain angiogenesis, we employ genetic approaches to inhibit astrogliogenesis during perinatal corticogenesis and examine its effects on brain vessel development. We find that conditional deletion from glial progenitors of *orc3*, a gene required for DNA replication, dramatically reduces glial progenitor cell number in the subventricular zone and astrocytes in the early postnatal cerebral cortex. This, in turn, results in severe reductions in both the density and branching frequency of cortical blood vessels. Consistent with a delayed growth but not regression of vessels, we find neither significant net decreases in vessel density between different stages after normalizing for cortical expansion nor obvious apoptosis of endothelial cells in these mutants. Furthermore, concomitant with loss of astroglial interactions, we find increased endothelial cell proliferation, enlarged vessel luminal size as well as enhanced cytoskeletal gene expression in pericytes, which suggests compensatory changes in vascular cells. Lastly, we find that blood vessel morphology in mutant cortices recovers substantially at later stages, following astrogliosis. These results thus implicate a functional requirement for astroglia in promoting blood vessel growth during brain development.

## Introduction

Astrocytes are one of the most abundant cell types in the brain. They not only play a key role in the normal development and function of the brain neural circuitry, but also intimately interact with the brain vascular network [1]. Astrocytes tightly ensheath blood vessels through their elaborate perivascular endfeet [2], and form a nearly complete covering of brain vessels [3], [4]. These interactions further enhance the blood-brain barrier (BBB) function of brain vascular cells. In addition, astrocytes are known to play an active role in mediating neurovascular coupling [5]. Several biochemical signals have been identified that are secreted by astrocytes and mediate astrocytic regulation of vasodilation or constriction. These findings thus indicate that astrocytes are not only structurally but also functionally linked to blood vessels in the brain. They also suggest that astrocytes may play a role in brain vascular network formation during development.

In the developing retina, blood vessel growth follows a meshwork laid down by astrocytic processes [6]. This suggests a role of astrocytes in regulating retinal angiogenesis. Indeed, recent studies show that perturbation of neuron-glial signaling can result in a hypoplastic astrocytic network and consequently impaired retinal vascular development, indicating functional involvement of astrocytes in this process [7]. Specific deletion of several classes of genes from retinal astrocytes has also been found to result in significant defects in retinal vessel development, indicating a multifaceted role played by astrocytes. This role so far includes secretion of vascular endothelial growth factor (VEGF) that promotes plexus spreading and endothelial cell proliferation and survival, assembly of a fibronectin matrix for endothelial filopodial alignment during retinal vessel growth, production of heparan sulfate proteoglycans that act synergistically with fibronectin in regulating VEGF distribution, and activation of extracellular matrix-bound latent ligands of the transforming growth factors β (TGFβ) family [8], [9], [10] (but also see [11], [12]). These results thus demonstrate a functionally critical role of astrocytes in regulating angiogenesis in the neural retina.

In the developing brain, astrocytes are first born during the perinatal period, at a time coincident with the onset of a period of extensive vascular network elaboration in the cerebral cortex [13], [14], [15]. Two main sources of progenitor cells have been identified that give rise to astrocytes in the cortex. One is radial glial cells that transform themselves into astrocytes near the pia during late embryogenesis, and the second is progenitor cells in the subventricular zone that continue to give birth to astrocytes during early postnatal development. Recent studies indicate that after populating the cortical plate, young astrocytes continue to proliferate locally, and that this may account for the rapid expansion of cortical astrocytes during the first three weeks of postnatal development [16]. Although they do not yet express proteins such as glial fibrillary acidic protein (GFAP) as mature astrocytes, young astrocytes in the early postnatal rat cortex have been found to interact closely with blood vessels by retroviral labeling [17]. Indeed, in these studies, analyses at both the light and electron microscopy level show that the contacts between astrocytes and vessels are not simply random encounters, but are complex interactions where the ends of astrocytic processes spread laterally along the vessels or curl around the periphery of vessels. These findings suggest that, as in the developing retina, astrocytes in the brain may also play a role in angiogenesis during early postnatal development. However, despite the well-known intimate interactions between astrocytes and blood vessels, especially in the adult brain, it remains, to date, undetermined whether astroglia play a role in regulating brain angiogenesis during development.

In this study, we employ a genetic approach of lineage specific cell cycle blockade to inhibit astroglial production in the perinatal cortex. We find that reduction of astroglia in the early postnatal cortex substantially delays vessel growth and branching, a phenotype associated with compensatory changes in both endothelial cells and pericytes. On the other hand, as gliosis occurs at later stages, vessel morphology substantially recovers. These results thus, for the first time, implicate a functionally significant role of astrocytes in promoting cortical vessel growth and branching during postnatal development, and lay the foundation for further elucidation of underlying molecular mechanisms.

## Results

### Interactions of astrocytes with blood vessels in the early postnatal mouse brain

**Figure 1:**
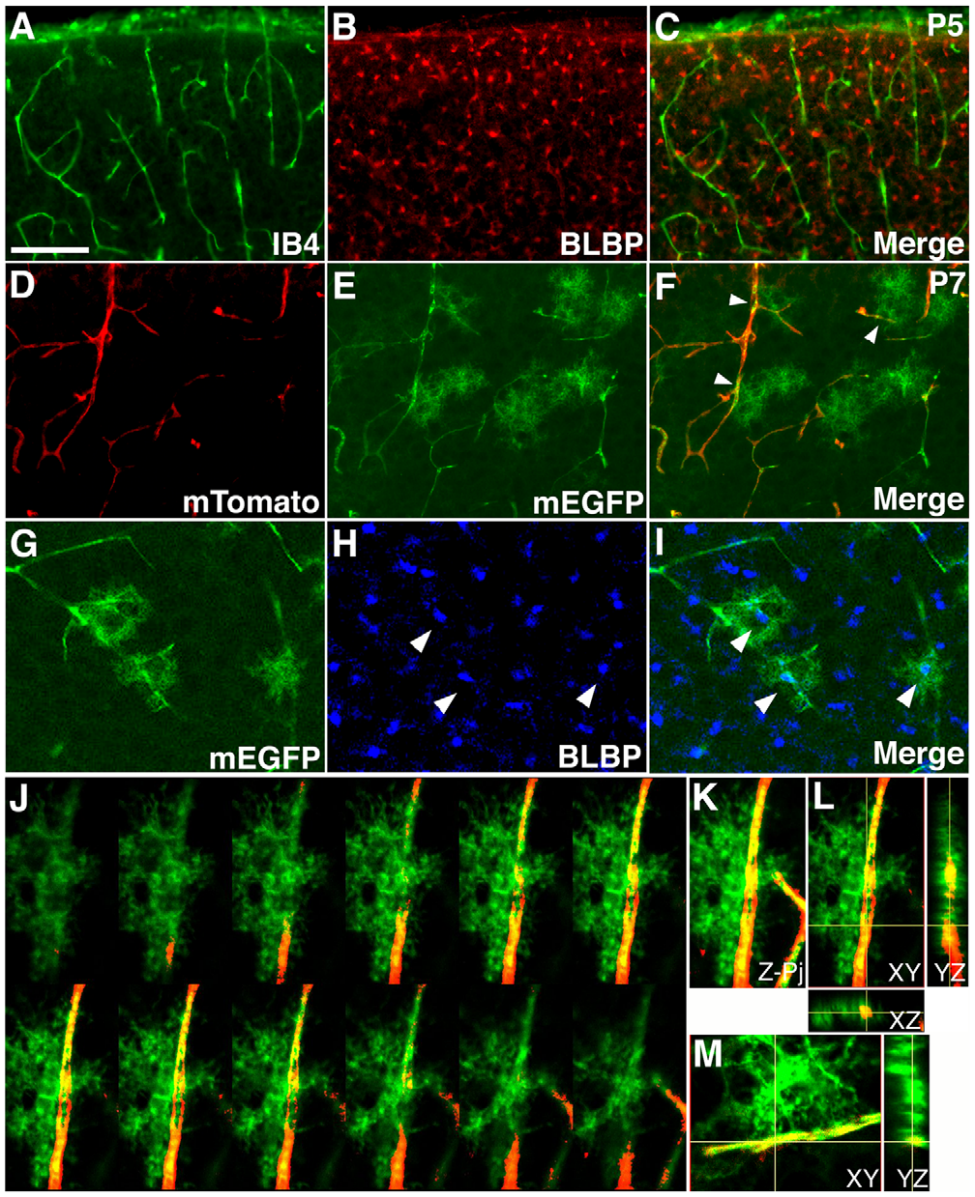
Close interactions between astrocytic processes and blood vessels in the early postnatal mouse cortex. (**A–C**) Limited interactions between the cell body of young astrocytes and blood vessels in the mouse cortex at P5. Blood vessels were stained using isolectin B4 (IB4, green in **A** & **C**), while young astrocytes were labeled using anti-BLBP antibodies (red in **B** & **C**). Limited overlap was observed between IB4 and BLBP staining. (**D–F**) Close interactions between astrocytic processes and blood vessel in the mouse cortex at P7. An *mTomato/mEGFP* reporter line was used to labeled astrocytes by *nestin-creER* mediated recombination induced at E18.0. A sporadic number of astrocyes and their processes were labeled by expression of mEGFP (green in **E** & **F**), while blood vessels continued to express a strong level of mTomato (red in **D** & **F**). The vast majorities of labeled astrocytes contact blood vessels though their processes (arrowheads in F). Also note green autofluorescence in blood vessels, which does not stain positive for anti-GFP antibodies. (**G–I**) Confocal microscopy analysis of mEGFP (green in **G** & **I**) and BLBP (blue in **H** & **I**) staining of brain sections as in (**D–F**). Each of the mEGFP positive cells overlaps with a single BLBP positive cell body (arrowheads in **H** & **I**), indicating that mEGFP positive cells are astrocytes. (**J–L**) Interactions between astrocytic processes and blood vessels examined by 3-D confocal reconstruction. Astrocytic processed are labeled by mEGFP (green). Blood vessels are labeled by mTomato (red). An astrocyte approaches from the left side and interact extensively with a blood vessel, with some processes wrapping around the vessel and interacting from the opposite side. A montage of selected Z-images is shown in (**J**), Z-projection in (**K**), and XY, XZ, and YZ cross-sections in (**L**). (**M**) Cross-sections of 3-D reconstruction of a second astrocyte that show clear interactions of individual astrocytic endfeet with a blood vessel. See supplemental information for movies of the 3-D reconstruction. Scale bar in (**A**), 200 μm for (**A–C**) and 100 for μm (**D–I**).

Previous studies using retroviral labeling have observed close interactions between astrocytes and blood vessels in the early postnatal rat cortex [17]. To determine whether similar interactions take place in the mouse brain, we examined astrocytes and blood vessels in the mouse cerebral cortex at postnatal day 5 (P5) (**Fig. 1**). To this end, we first stained wildtype P5 brain sections with isolectin B4 (IB4) to identify blood vessels, and anti-brain lipid binding protein (BLBP) antibodies to identify young astrocytes that transiently express BLBP after birth [16] (**Fig. 1A–C**). In these sections, we found a large number of BLBP positive young astrocytes scatter across the cortical plate at P5 (**Fig. 1B**). However, since anti-BLBP antibodies most strongly stain astrocytic cell bodies, no preferential overlap was obvious between astrocytes and blood vessels (**Fig. 1C**). This suggests that astrocytes may interact with blood vessels predominantly through their processes.

To visualize astrocytic processes, we next employed an inducible *nestin-creER* line [18], together with an *mTomato/mEGFP* reporter that expresses membrane-bound mTomato before and membrane-bound mEGFP after *cre* induced recombination [19] (**Fig. 1D–F** **'**). Previous studies have shown that tamoxifen administration during late embryogenesis specifically labels cortical astrocytes [18]. Indeed, we found that administration of tamoxifen around embryonic day 18.0 (E18.0) induced sporadic labeling of cortical cells with the characteristic morphology of astrocytes at P7 (**Fig. 1E**). The blood vessels, on the other hand, continue to express a strong level of mTomato (**Fig. 1D**). When double labeled with anti-BLBP antibodies, the EGFP positive cells were invariably positive for BLBP at their cell bodies (**Fig. 1G–I**). This indicates that they are indeed astrocytes. In these sections, we found that all labeled astrocytes had a bushy morphology and the vast majorities were in contact with blood vessels (**Fig. 1F**). The astrocytic processes also appeared overwhelmingly confined to one side of the blood vessels (arrowheads in **Fig. 1F**). Indeed, 3-dimensional reconstruction by confocal microscopy showed that the astrocytes consistently approached blood vessels from one side and interacted with vessels through multiple processes (**Fig. 1J–M**, **Movies S1, S2**). In cases where it appeared, under conventional microscopy, that the astrocytes wrapped around a vessel, 3-dimensional reconstruction showed that the astrocytes were in fact located either in front of or behind the blood vessels. Although sometimes astrocytes were also observed to send processes wrapping around a vessel and interacted with it from the opposite direction, the number of these processes tended to be small (**Fig. 1J–L**). Thus, it appears likely that the same segment of a blood vessel may simultaneously interact with processes from multiple astrocytes. Altogether, these results indicate that, similar to the rat cortex, astrocytes in the early postnatal mouse cortex also interact closely with blood vessels.

### Inhibition of cortical astrogliogenesis by conditional *orc3* gene deletion

**Figure 2:**
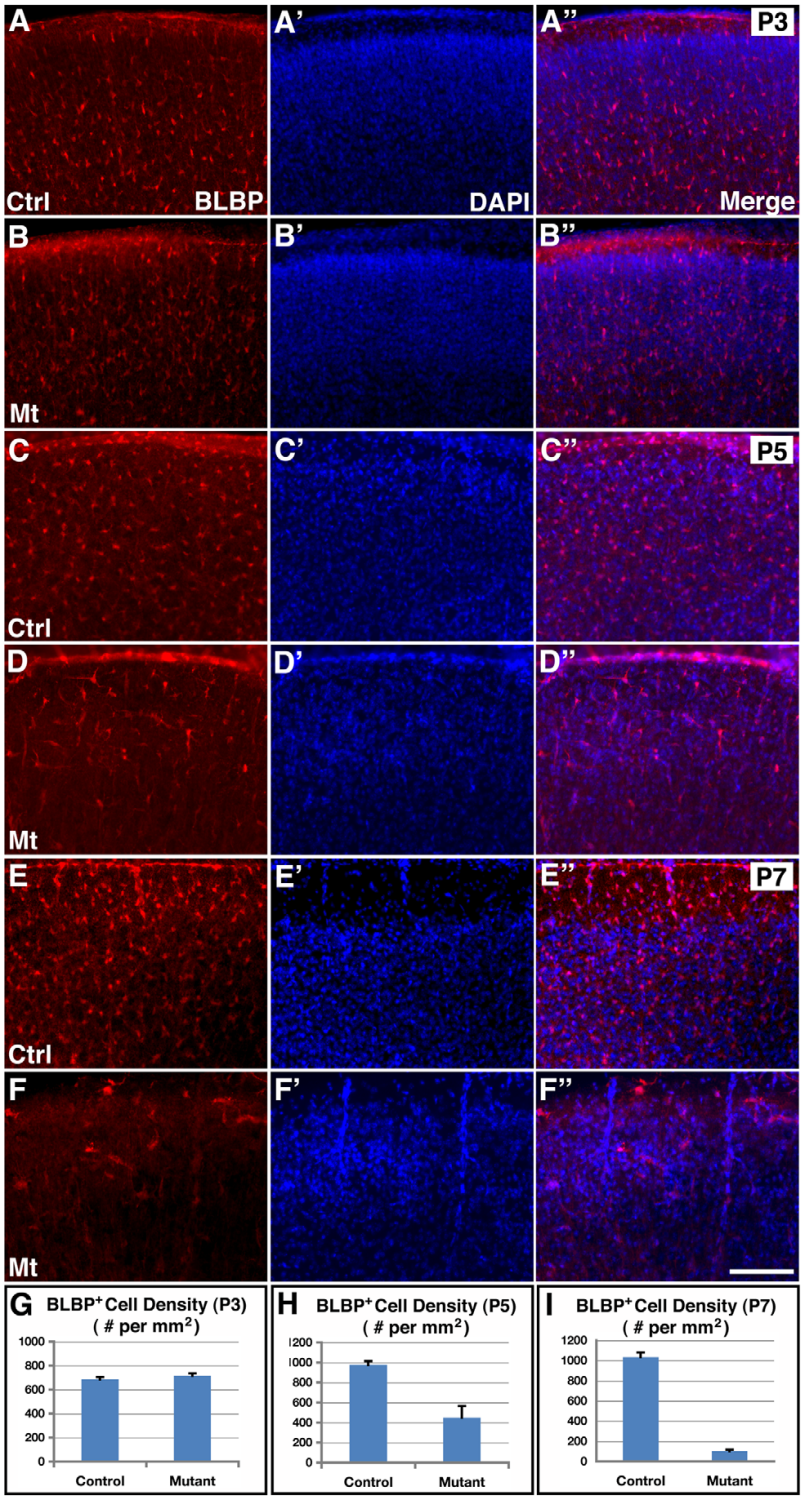
Inhibition of cortical astrogliogenesis by *hGFAP-cre* mediated *orc3* gene deletion in class II mutants. (**A–F”**) Young astrocytes were stained using anti-BLBP antibodies (red in **A–F** and **A”–F”**), and nuclei were labeled by DAPI staining (blue in **A'–F'** and **A”–F”**) in control and *orc3* mutant cortices at P3 (**A–B”**), P5 (**C–D”**) and P7 (**E–F”**). No obvious differences were observed in the density of BLBP positive astrocytes between control (**A–A”**) and mutant (**B–B”**) cortices at P3. However, obvious reductions were observed in mutants (**D–D”**) as compared to controls (**C–C”**) at P5. Young astrocytes also appeared more elongated in the mutant cortex at P5. By P7, BLBP positive astrocytes were almost completely eliminated from mutants (**D–D”**) as compared to controls (**C–C”**). (**G–I**) Quantification of BLBP positive cell density in P3 (**G**), P5 (**H**), and P7 (**I**) control and mutant cortices. Statistical analysis by Student's *t* test showed no significant changes in mutants at P3 (control, 685.6±19.1/mm^2^; mutant, 716.0±18.8/mm^2^; *P* = 0.27, n = 9), but significant decreases in BLBP positive cell density at both P5 (control, 974.8±38.6/mm^2^; mutant, 447.7±117.4/mm^2^; *P* = 0.001, n = 11) and P7 (control, 1033±47/mm^2^; mutant, 701±14/mm^2^; *P* = 0.0001, n = 4). Scale bar in (**F”**), 200 μm for (**A–F**).

The *orc3* gene encodes a core subunit of an origin recognition complex (ORC) essential for DNA replication from yeast to human cells [20]. Together with 5 other ORC subunits, it forms a protein complex involved in the first step of DNA replication, by binding to DNA replication origins and facilitating the recruitment of additional DNA replication factors and polymerases. Since all six subunits are essential for the formation of ORC, removal of any subunit will compromise complex function. We have generated a conditional allele of the *orc3* gene by flanking exons 5–7 with a pair of loxP sites (**Fig. S1**). This therefore provides an opportunity to ablate specific cell types through blocking lineage specific progenitor cell proliferation. To inhibit astroglial production, we employed an *hGFAP-cre* line that targets cortical neural progenitors during mid to late embryogenesis [21]. We found that introduction of *hGFAP-cre* into a background of homozygous *orc3* conditional alleles resulted in two classes of mutants (**Table 1**) (**Fig. S2**). In the first group, we observed consistent midline hemorrhage in the cortex during the perinatal period (P0-3), which then spreads to neighboring regions at later stages (P5–7). In this group, we also consistently observed cortical degeneration after P5. By contrast, in the second group, we observed no obvious brain hemorrhage or cortical degeneration throughout the postnatal period. We hypothesized that these distinct phenotypes may result from differences in the efficiency of *hGFAP-cre* mediated *orc3* gene deletion. Indeed, when we examined cell proliferation in subventricular zone at P3, we found a severe reduction in Ki67 positive cells in class I mutants, in comparison to an intermediate reduction in class II mutants (**Fig. S3**). As a result, when stained with anti-BLBP antibodies, all of the class I mutants we examined at P0-3 showed severe defects in cortical astrocyte production (**Fig. S4A–D**). By contrast, none of the four class II mutants we examined showed obvious reductions at the same stages (data not shown, see also Fig. 2A–B” & 2G below). This indicates that *orc3* gene deletion takes place earlier in class I than in class II mutants. In further support of our classification of the mutants, we also observed a consistent ratio of roughly 2:1 between class I and class II mutants throughout postnatal development (**Table 1**). Since we observed no premature lethality before P21, these findings further support the interpretation that the distinct phenotypes in class I and class II mutants likely result from differences in the efficiency of *hGFAP*-*cre* mediated recombination during embryogenesis, when, unlike class II mutants, the prospective class I mutants have undergone significant recombination. As a result, the severe loss of cortical astrocytes in class I mutants may have led to the severe vascular defects at an early postnatal stage (**Fig. S4E–H**), as well as consequent cortical degeneration [22], [23]. Although we observed severe defects in vessel development in class I mutants at P3 (**Fig. S4 E–H**), which supports the interpretation of a critical role of astrocytes in promoting postnatal brain vessel development, however, the cortical degeneration at subsequent stages precludes further analysis of brain angiogenesis. For this reason, we focused our analysis on class II mutants.

**Table 1:** Classification of *orc3/hGFAP-cre* mutant postnatal phenotype.

	P0-3		P5–7		P14/15		P21	
Class of mutants	I	II	I	II	I	II	I	II
Number of mutants	13	8	34	17	14	8	2	1
Midline hemorrhage	10/13	0/8	3/16	0/10		0/8		0/1
Widespread hemorrhage	3/13	0/8	13/16	0/10		0/8		0/1
Cortical degeneration			16/16	0/10	14/14	0/8	2/2	0/1
Reduced astrocyte density	4/4	0/4		3/3		3/3		

To thoroughly determine effects of *orc3* deletion on cortical astrogliogenesis in class II mutants, we next employed anti-BLBP antibodies to stain young astrocytes at P3, P5, and P7 (**Fig. 2**). At P3, we found a dense array of BLBP positive astrocytes scatter across the cortical plate in both controls and mutants and there were no obvious differences in the distribution of astrocytes between these two genotypes (**Fig. 2A–B** **”**). At P5, we observed a similar pattern of BLBP positive cell distribution in the control cortex as at P3. However, the number of astrocytes in P5 mutants appeared reduced and their morphology appeared more elongated (**Fig. 2C–D** **”**). By P7, although the control cortex still showed a similar dense array of BLBP positive astrocytes, their numbers appeared further reduced in mutants, where only a few BLBP positive cells could be found sporadically across the cortical plate (**Fig. 2E–F** **”**). Quantification showed that while the density of young astrocytes was not significantly altered at P3 (control, 686±19/mm^2^; mutant, 716±19/mm^2^; *P* = 0.27, n = 9), it was severely reduced, by about 54%, in mutants at P5 (control, 975±39/mm^2^; mutant, 448±117/mm^2^; *P* = 0.001, n = 11), and near completely eliminated, by about 90%, at P7 (control, 1033±47/mm^2^; mutant, 101±14/mm^2^; *P* = 0.0001, n = 4) (**Fig. 2G** **–**). These results thus indicate that *orc3* deletion in class II *orc3/hGFAP-cre* mutants significantly inhibits cortical astrogliogenesis beginning at P5 and severely blocks this process by P7.

**Figure 3:**
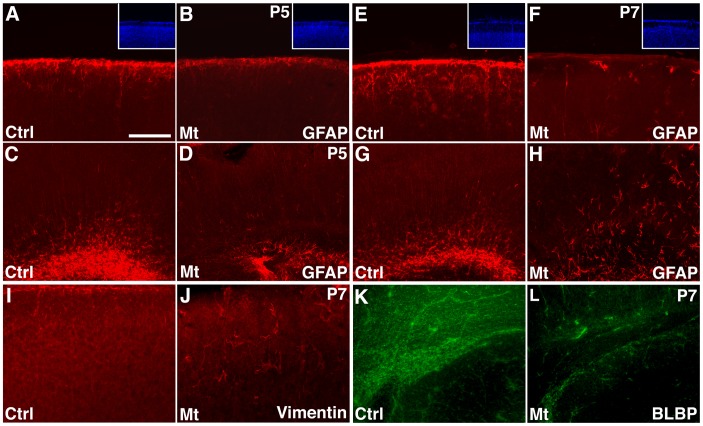
Reduced glial progenitors in the subventricular zone following *hGFAP-cre* mediated *orc3* gene deletion in class II mutants. (**A–H**) Decreased numbers of astrocytes near the pia as well as glial progenitors in the subventricular zone in *orc3* mutant cortices at P5 (**A–D**) and P7 (**E–H**). Astrocytes and glial progenitors in control (**A, C, E, & G**) and mutant (**B, D, F, & H**) cortices were identified by staining with anti-GFAP antibodies. See quantification in text. DAPI staining showed that the pia surface was intact in both control and mutant sections (insets in (**A, B, E, F**)). (**I–J**) Reduced numbers of vimentin positive astrocytes near the pia in the mutant cortex (**J**) as compared to controls (**I**) at P7. (**K–L**) Dramatic reduction in the number of BLBP positive progenitor cells in the subventricular zone in mutants (**L**) as compared to controls (**K**) at P7. Scale bar in (A), 200****μm for (**A–L**).

To further evaluate effects of *orc3* deletion on astrogliogenesis, we next examined GFAP staining in the cortex at P5 and P7 (**Fig. 3**). At these stages, GFAP staining identifies two main cell populations in the wildtype cortex. One is astrocytes near the pia that derive from radial glia during the perinatal period, and the second is progenitor cells in the subventricular zone that serve as another source of postnatal astrocytes. In contrary to these cells, young astrocytes in the cortical plate at these stages show minimal GFAP protein expression. We found that at both P5 and P7, while there was obvious clustering of GFAP positive cells near the pia in controls, their numbers were significantly reduced in mutants (**Fig. 3A, 3B, 3E, and 3F**). The reduced GFAP staining was unlikely due to disruption of the cortical pia since DAPI nuclear staining revealed a relatively intact layer of meningeal cells (**Fig. 3A, 3B, 3E, and 3F** insets). In addition, in mutants, we also observed a reduction in the staining of Vimentin, another marker for radial glial progenitors and astrocytes, in the same regions of the cortex (**Fig. 3I and 3J**). This indicates that *orc3* deletion significantly inhibits the production of astrocytes near the pia. Similarly, at the subventricular zone, while we observed strong GFAP staining in controls at both P5 and P7, the number of GFAP positive cells was severely reduced at both stages (**Fig. 3C, 3D, 3G, and 3H**). Quantification of GFAP staining intensity showed a 46% reduction at P5 (control, 1986±60 AU (Arbitrary Units); mutant, 1070±75 AU; *P* = 5.1×10^−7^, n = 8) and a 53% reduction at P7 (control, 2388±141 AU; mutant, 1131±40 AU; *P* = 0.0001, n = 4) in mutants as compared to controls of the same stage. Moreover, we also observed a dramatic loss of BLBP staining in the mutant subventricular zone at P7 (**Fig. 3K and 3L**). By contrast, in the cortical plate, there was an apparent increase in the number of GFAP positive cells in the mutants, especially at P7 (Fig. 3G–H). This suggests that radial glial depletion at this stage may also compromise the migration of astrocyte precursors out of the subventricular zone. In addition, Vimentin staining around vessels in mutants appeared increased (**Fig. 3I–J**), suggesting potential compensatory changes in the mutant vasculature. Thus, these results together indicate that *orc3* deletion by *hGFAP-cre* not only severely blocks radial glial transformation into astrocytes at the pia but also depletes glial progenitors in the subventricular zone. *hGFAP-cre* mediated *orc3* gene deletion is therefore an efficient genetic approach for inhibiting postnatal cortical astrogliogenesis.

### Effects of *orc3* gene deletion on cortical neurogenesis and oligogenesis

**Figure 4:**
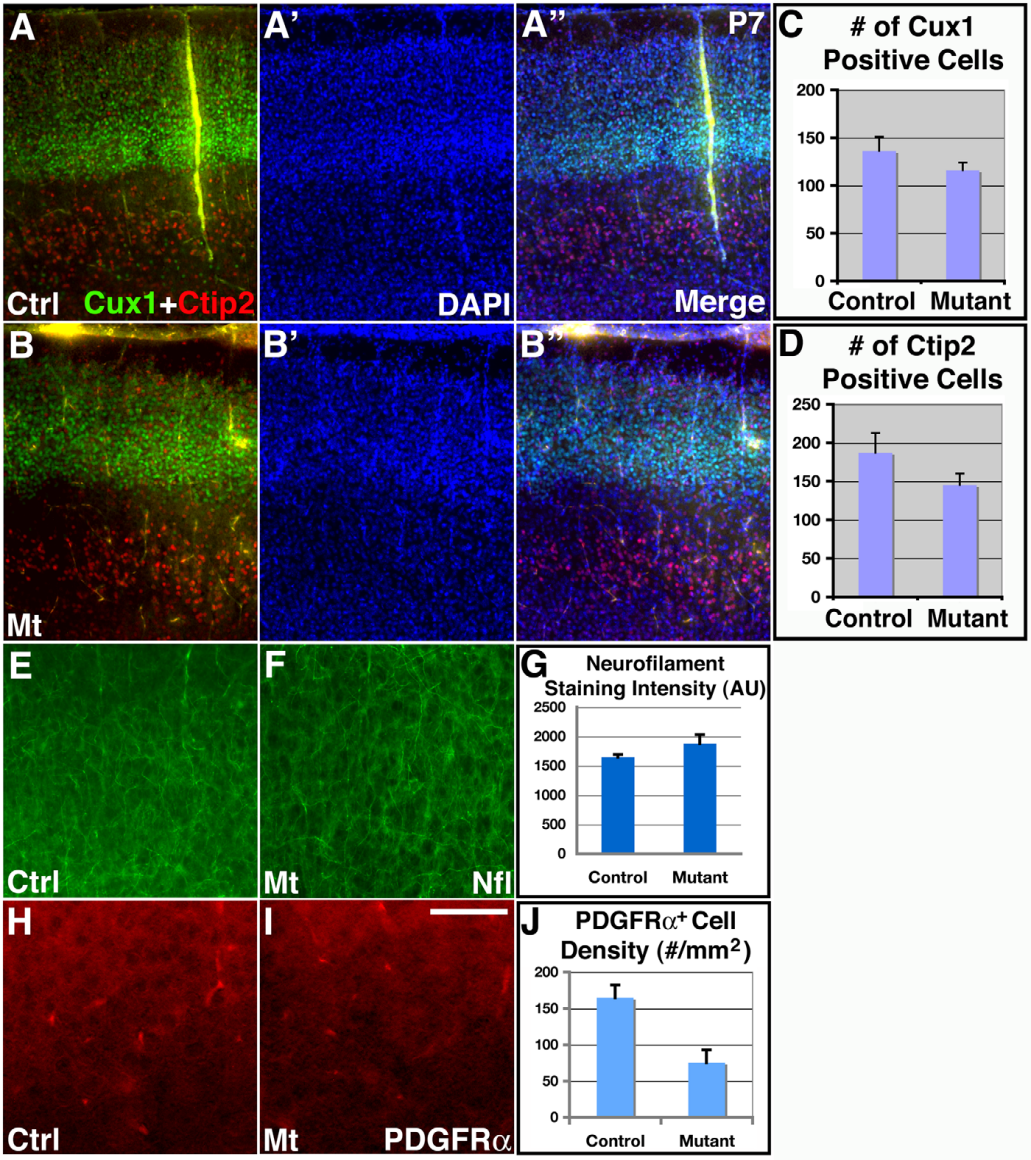
Relatively normal neurogenesis and neuronal differentiation in class II *orc3/hGFAP-cre* mutant cortices. (**A–B”**) Normal expression of layer specific neuronal markers as well as relatively normal number of neurons in *orc3* mutant cortices at P7. Upper layer neurons were stained using ant-Cux1 antibodies (green in **A**, **A”**, **B** & **B”**), while lower layer neurons were stained using anti-Ctip2 antibodies (red in **A**, **A”**, **B** & **B”**). No obvious differences were observed in the numbers of Ctip2 and Cux1positive neurons between controls (**A–A”**) and mutants (**B–B”**). (**C–D**) Quantification of Cux1 and Ctip2 positive neurons in control and mutant cortices. No significant differences were observed between controls and mutants in either the number of Cux1 positive cells per cortical width of 77 μm (control, 131.1±14.8; mutant, 115.9±8.2; *P* = 0.26; n = 7), or the number of Ctip2 positive cells per cortical width of 660 mm (control, 187.0±25.7; mutant, 145.3±14.6; *P* = 0.22; n = 4). (**E–G**) Normal neuronal differentiation in *orc3* mutant cortices at P7 as assessed by anti-neurofilament staining. No obvious differences were observed between controls (**F**) and mutants (**E**). Quantitative analysis also revealed no statistically significant differences (control, 1644±47; mutant, 1877±156; *P* = 0.23, n = 3) in staining intensity (AU, arbitrary unit) (**G**). (**H–J**) Reduced oligodendrocyte production in *orc3* mutant cortices at P7. PDGFRα staining revealed reduced numbers of positive cell bodies in mutants (**I**) as compared to controls (**H**). Quantification confirmed reductions in PDGFRa positive cell body density in mutants (control, 164.8±17.4; mutant, 75.3±17.6; *P* = 0.002, n = 12) (**J**). Scale bar in (**I**), 200 μm for (**A–B'**) and (E–F), 50 μm for (**H–I**).

*hGFAP-cre* targets cortical radial glial progenitors during mid to late embryonic corticogenesis [21], [24]. To determine effects of *orc3* deletion on cortical neurogenesis, we next stained brain sections with several layer specific neuronal markers (**Fig. 4A–B** **”**). First, we used an anti-Ctip2 antibody to label neurons of the deep cortical layers. We found that at P7, anti-Ctip2 antibodies labeled a similar number of cells in controls and mutants. Indeed, quantification showed no significant differences in the number of Ctip2 positive cells between these two genotypes (control, 187.0±25.7; mutant, 145.3±14.6/cortical width of 660****μm; *P* = 0.22; n = 4) (**Fig. 4D**). This indicates that *orc3* deletion by *hGFAP-cre* does not significantly affect the production of deep layer neurons, consistent with their early birthdates. Next, we employed an anti-Cux1 antibody to label neurons of the upper cortical layers. We found that, although the Cux1 positive layer appeared slightly thinned, the vast majority of Cux1 positive neurons were properly produced and all were localized normally to the upper cortical laminae. Indeed, quantification also revealed no significant differences in Cux1 positive cells (control, 131.1±14.8; mutant, 115.9±8.2/cortical width of 77 μm; *P* = 0.26; n = 7) (**Fig. 4C**). The apparent thinning of Cux1 positive layer may thus result, at least in part, from a reduced number of astrocytes in the same brain region. Thus, these results indicate that *orc3* deletion by *hGFAP-cre* does not dramatically compromise cortical neurogenesis.

To evaluate potential defects in cortical neuron differentiation, we examined neurofilament expression at P7 (**Fig. 4E–G**). Neurofilaments are intermediate filament proteins expressed in neuronal processes as neurons undergo terminal differentiation. We found that at P7, neurons in the control and mutant cortices expressed a similar level of neurofilament (**Fig. 4E and 4F**). Quantification also showed that neurofilament staining intensity was not significantly different between the two genotypes (control, 1644 ± 47 AU; mutant, 1877±156 AU; *P* = 0.23, n = 3) (**Fig. 4G**). Thus, these results indicate that *orc3* deletion by *hGFAP-cre* also does not obviously affect cortical neuron differentiation. Lastly, to evaluate potential increases in cell death, we employed an antibody against active caspase 3 to determine the number of apoptotic cells. We found that, although there were occasional cells that stained positive for active caspase 3 in the mutant cortex (between 0–3 cells per field at 10x magnification), their numbers were similar to controls, and were also minimal in comparison to the large number of neurons present in the cortical plate (**Fig. S5**). Thus, these results indicate that neither is there a dramatic increase in apoptosis. Thus, these results indicate that *orc3* deletion by *hGFAP-cre* largely spares neurogenesis and neuronal survival and differentiation in the cerebral cortex.

Three waves of oligodendrocyte production have been identified that populate the cerebral cortex in a sequential manner, two of which originate from the ventral forebrain while the third wave arises within the postnatal cerebral cortex itself [25]. To determine whether *orc3* deletion by *hGFAP-cre* affects cortical oligodendrocyte development, we next stained brain sections with an antibody against PDGFRα, a well-known marker for cortical oligodendrocyte precursors [25]. Since PDGFRα also stains astrocyte endfeet, we focused on stainings that resembled cell bodies. We found that at P7, *orc3* deletion resulted in a significant reduction in the number of PDGFRα positive cell bodies throughout the cortical plate (**Fig. 4F and 4G**). Quantification showed that the density of PDGFRα positive cell bodies was reduced by about 50% in mutants at P7 (control, 165±17/mm^2^; mutant, 75±18 /mm^2^; *P* = 0.002, n = 12) (**Fig. 4H**). This suggests a reduction in the number of oligodendrocyte precursors in the mutant cortex. This reduction, however, is significantly smaller than that observed for astrocytes at P7 (∼90% reduction) (**Fig. 2I**). This may be due to the three waves of forebrain oligodendrocyte production, which have been shown to compensate for each other during development [25]. Thus, these results indicate that *orc3* deletion by *hGFAP-cre* **may** also result in a moderate defect in oligodendrocyte production in the cortex.

### Effects of inhibiting astrogliogenesis on cortical vessel development

**Figure 5:**
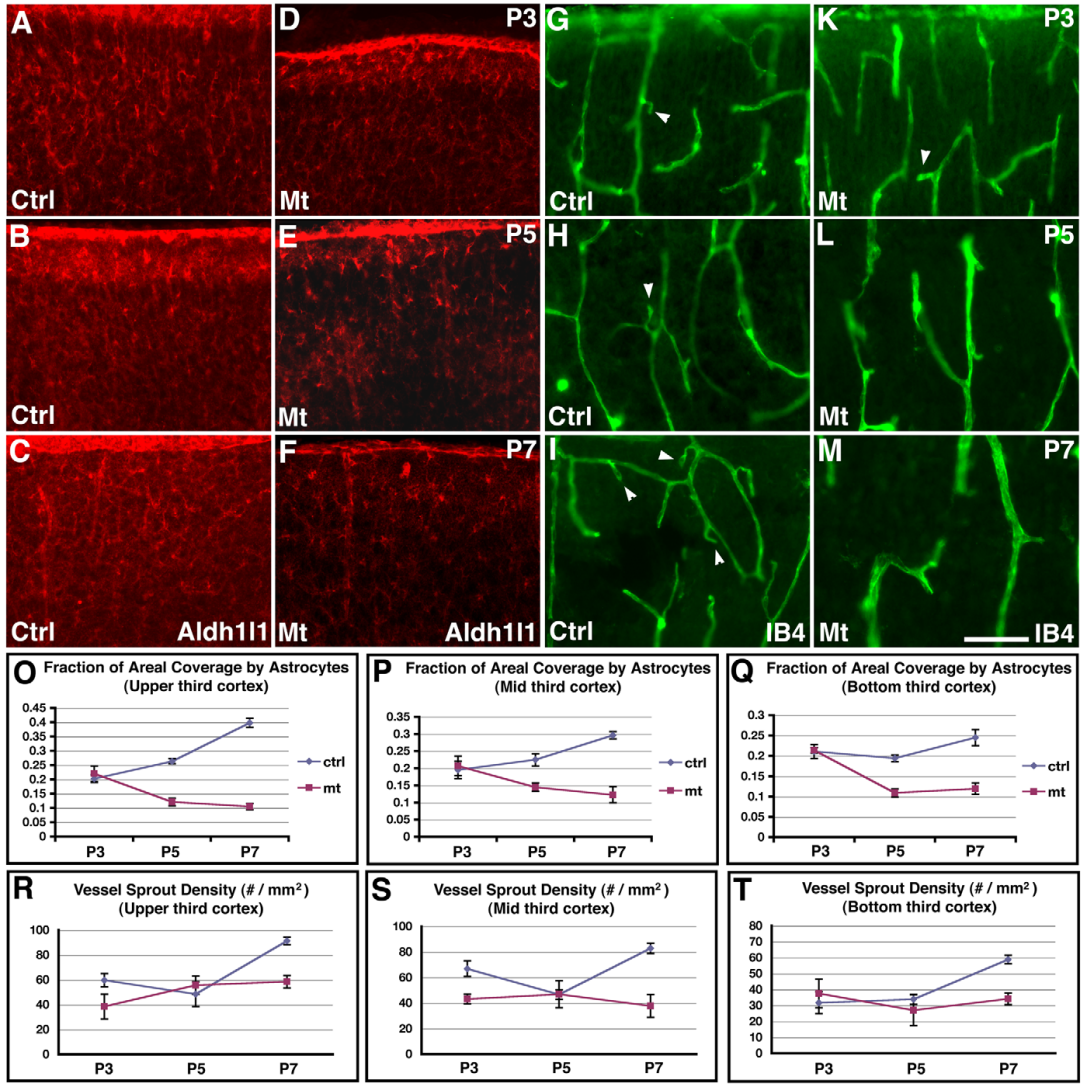
Patterns of astrocytic process coverage and vessel sprouting in the control and class II *orc3/hGFAP-cre* mutant cortices during early postnatal development. (**A–C**) Astrocytic process coverage in the upper third of control cortices at P3 (**A**), P5 (**B**), and P7 (**C**). Astrocytic processes were stained using an anti-Aldh1l1 antibody. Quantification (**O**) revealed small but significant increases in coverage from P3 to P5 and from P5 to P7. (**D–F**) Astrocytic process coverage in the upper third of class II mutant cortices at P3 (**D**), P5 (**E**), and P7 (**F**). Quantification (**O**) revealed a small but significant reduction in coverage from P3 to P5, which remains unchanged from P5 to P7. (**G–I**) Vessel sprouting in the upper third of control cortices at P3 (**G**), P5 (**H**), and P7 (**I**). Vessels were stained using IB4. Quantification (R) revealed no changes in sprout density from P3 to P5, but a significant increase from P5 to P7. (**J–L**) Vessel sprouting in the upper third of class II mutant cortices at P3 (**J**), P5 (**K**), and P7 (**L**). Quantification (R) revealed no significant changes in sprout density either from P3 to P5 or from P5 to P7. (**O–P**) Quantification of astrocytic process coverage in the upper (**O**), mid (**P**), and bottom (**Q**) thirds of the control (blue circles) and class II mutant (red squares) cortex at P3, P5 and P7. Similar trends of increases in coverage from P3 to P5 and from P5 to P7 were observed in all three sectors in controls, although the changes are statistically significant only in the upper sector. In mutants, astrocytic coverage tends to decrease from P3 to P5 and then remains flat from P5 to P7, although the early decreases are statistically significant only in the upper and bottom sectors. (**R–T**) Quantification of vessel sprout density in the upper (**R**), mid (**S**), and bottom (**T**) thirds of the control (blue circles) and class II mutant (red squares) cortex at P3, P5 and P7. Similar trends of increases in sprout density from P5 to P7 were observed in all three sectors in controls, although the changes are statistically significant only in the upper and middle sectors. In mutants, sprout density remains flat throughout the period from P3 to P7. Scale bar in (**M**), 100****μm for (**A–M**).

Our results so far indicate that *orc3* deletion by *hGFAP-cre* results in a relatively specific inhibition of cortical astrogliogenesis in the early postnatal cortex. This provides a unique opportunity to evaluate the functional involvement of astrocytes in postnatal brain angiogenesis. To this end, we first examined patterns of astrocytic process coverage of the postnatal cortex using an anti-Aldh1l1 antibody, and analyzed patterns of vessel sprouting by IB4 staining (**Fig. 5**). To detect potential spatial variations in these patterns, we also divided the cortex into three sectors: upper, middle, and bottom thirds. Since unlike in the retina, astrocytic process growth and vessel sprouting in the cortex take place in three dimensions, the above approach is likely only to capture part of the potential patterns. Nonetheless, it may still reveal significant information. Indeed, we found that in controls, all three sectors show similar trends of increases in astrocytic coverage from P3 to P5 and from P5 to P7, although the changes between these neighboring stages are statistically significant only in the upper third of the cortex (P3, 20.24±1.35%; P5, 26.33±0.88%; P7, 39.81±1.54%; *P* = 0.44 between P3 and P5; *P*<0.001 between P5 and P7; n = 6) (**Fig. 5O–Q**, blue circles). Concomitantly, we found similar trends of increases in sprout density from P5 to P7 in all three sectors in controls, although the changes are statistically significant only in the upper and middle sectors (upper sector: P5, 48.50±2.90/mm^2^; P7, 91.33±3.99/mm^2^; *P*<0.001; n = 6; middle sector: P5, 46.67±3.84/mm^2^; P7, 82.83±5.98 /mm^2^; *P* = 0.010; n = 6) (**Fig. 5R–T**, blue circles). Further analysis showed that the correlation coefficient between astrocytic coverage and sprout density is 0.75 for the entire cortex. This indicates that there is a close relationship between astrocytic process growth and vessel sprouting during the early postnatal period, supporting the interpretation of a role of astrocytes in promoting vessel growth. By contrast, in class II *orc3/hGFAP-cre* mutants, astrocytic coverage tends to decrease from P3 to P5 and then remains unchanged from P5 to P7, although the early decreases from P3 to P5 are statistically significant only in the upper and bottom sectors (upper sector: P3, 22.00±2.74%; P5, 12.09±1.33%; *P* = 0.001; n = 6; bottom sector: P3, 21.38±0.70%; P5, 10.89±0.98%; *P*<0.001; n = 5) (**Fig. 5O–Q**, red squares). As a consequence, sprout density remains unchanged in the mutants throughout the entire period from P3 to P7 in all three sectors (**Fig. 5R–T**, red squares). These results thus further indicate that astrocytes play a key role in functionally promoting vessel sprouting during the early postnatal period.

**Figure 6:**
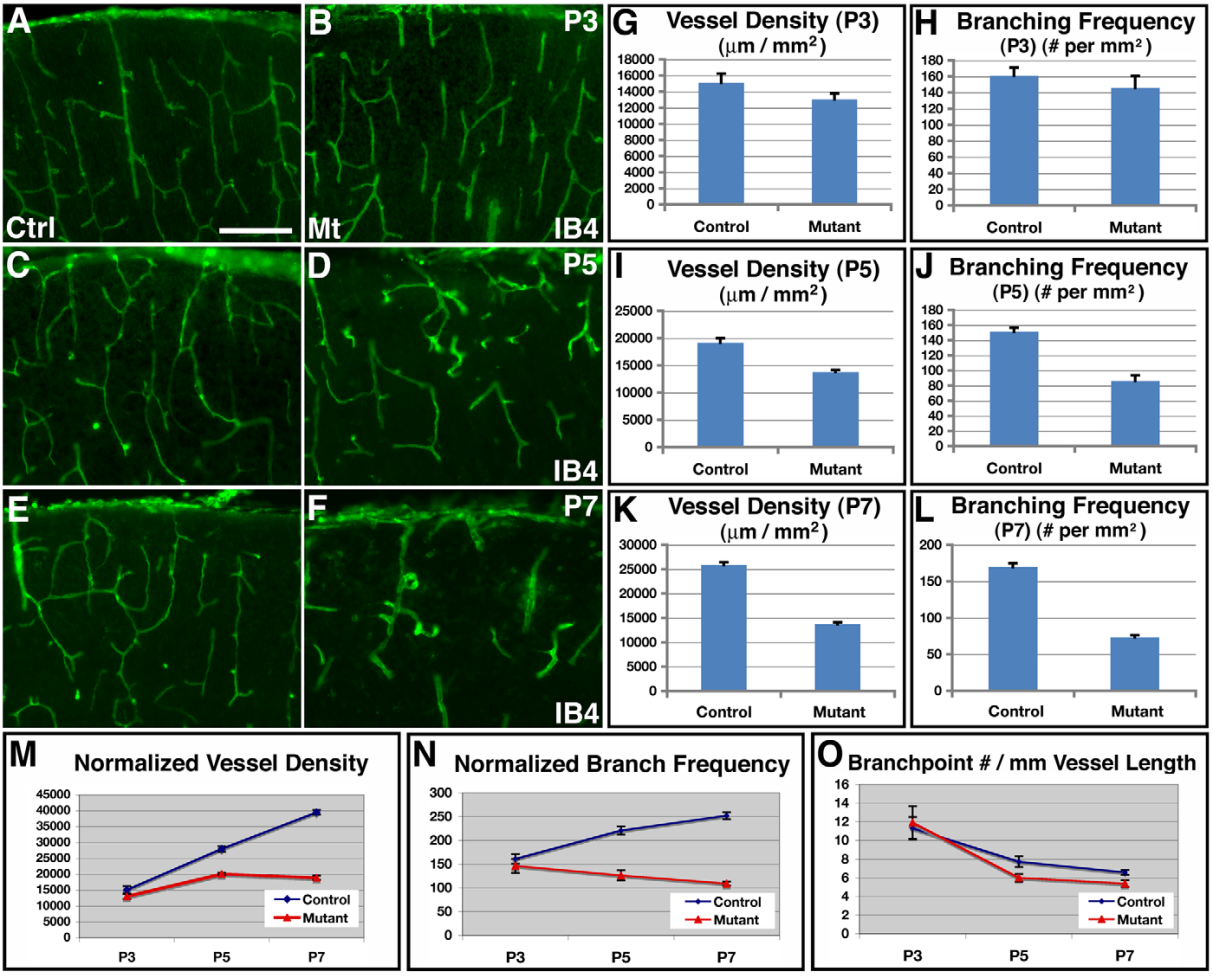
Defective vessel development following reduced astrogliogenesis in class II *orc3/hGFAP-cre* mutant cortices. (**A–F**) Vessel morphology, as revealed by IB4 staining, in control (**A, C & E**) and mutant (**B, D & F**) cortices at P3 (**A & B**), P5 (**C & D**), and P7 (**E & F**). No obvious differences in vessel development were observed between controls and mutants at P3. Areas with reduced vessel density were frequently observed in mutants at P5. Obvious defects in vessel development were observed in mutants at P7. (**G–L**) Quantification of vessel density and branch point frequency in control and mutant cortices at P3 (**G & H**), P5 (**I & J**), and P7 (**K & L**). Statistical analysis by Student' *t* test showed that there were no significant differences in either vessel density (control, 15109±1139 μm/mm^2^; mutant 13027±753 μm/mm^2^; *P* = 0.14, n = 9) or branch point frequency (control, 160.9±10.2/mm^2^; mutant 146.0±14.6/mm^2^; *P* = 0.42, n = 9) at P3, but significant reductions in both at P5 (vessel density: control, 19134±849 μm/mm^2^; mutant 13754±408 μm/mm^2^; *P* = 1.7×10^−5^, n = 14; branching frequency: control, 151.0±5.7/mm^2^; mutant 86.2±7.4/mm^2^; *P* = 4.8×10^−6^, n = 9), as well as at P7 (vessel density: control, 25909±487 μm/mm^2^; mutant 13704±402 μm/mm^2^; *P* = 1.8×10^−6^, n = 4; branching frequency: control, 169.8±4.9/mm^2^; mutant 73.2±3.1/mm^2^; *P* = 1.4×10^−5^, n = 4). (**M–O**) Quantitative analysis of normalized vessel growth and branching from P3 to P7 in control and mutant cortices. Vessel density and branching frequency were normalized against cortical area expansion (**M &N**). Branching frequency was normalized against total vessel length (**O**). In control brains, normalized vessel density significantly increased during the periods both from P3 to P5 (*P* = 9.5×10^−8^, n = 11) and from P5 to P7 (*P* = 2.1×10^−6^, n = 12). By contrast, in mutants, although normalized vessel density also increased from P3 to P5 (*P* = 10^−6^, n = 9), it grew at a slower rate. Moreover, there were no significant changes from P5 to P7 (*P* = 0.77, n = 12). Similarly, in controls, normalized branching frequency increased significantly during the periods both from P3 to P5 (*P* = 0.0003, n = 10) and from P5 to P7 (*P* = 0.019, n = 9). By contrast, in mutants, it remained not significantly changed during the period either from P3 to P5 (*P* = 0.29, n = 9) or from P5 to P7 (*P* = 0.17, n = 9). Scale bar in (**A**), 200 μm for (**A–H**).

To further characterize effects of glial depletion on brain angiogenesis, we examined overall vascular network morphology from P3 to P7 (**Fig. 6A–F**). At P3 in controls, blood vessels form a dense network that more or less evenly populate the entire cortical plate (**Fig. 6A**). A similar vascular network was also observed in mutants at this stage (**Fig. 6B**). At P5 in the control brain, we observed a similarly dense vascular network as at P3 (**Fig. 6C**). However, in mutants, we frequently observed areas with obvious reductions in vessel coverage (**Fig. 6D**). By P7, these deficits have become more severe. In contrast to the dense vessel network observed in the control cortex (**Fig. 6E**), IB4 positive vessels were only sparsely observed in mutants at P7and they appeared severely enlarged (**Fig. 6F**). Quantitative analysis showed that, while the cortical vessel density was not significantly different between controls and mutants at P3 (control, 15109±1139 μm/mm^2^; mutant, 13027±753 μm/mm^2^; *P* = 0.14, n = 9) (**Fig. 6G**), the density was significantly reduced, by about 28%, in mutants at P5 (control, 19135±849 μm/mm^2^; mutant, 13745±408 μm/mm^2^; *P* = 1.7×10^−5^, n = 14) (**Fig. 6I**), and dramatically lower, by about 47%, at P7 (control, 25909±487 μm/mm^2^; mutant, 13704±402 μm/mm^2^; *P* = 1.8×10^−6^, n = 4) (**Fig. 6K**). Similarly, while not significantly affected at P3 (control, 161±10/mm^2^; mutant, 146±15/mm^2^; *P* = 0.42, n = 9) (**Fig. 6H**), the vessel branch point frequency was also severely reduced in mutants, by about 43%, at P5 (control, 151±6 /mm^2^; mutant, 86±7/mm^2^; *P* = 4.8×10^−6^, n = 9) (**Fig. 6J**) and by about 57%, at P7 (control, 170±5/mm^2^; mutant, 73±3/mm^2^; *P* = 1.4×10^−5^, n = 4) (**Fig. 6L**). Thus, these results provide additional strong support for the conclusion that inhibition of astrogliogenesis by *hGFAP-cre* mediated *orc3* deletion results in severe deficits in cortical vessel development.

To determine the mechanisms underlying the vascular phenotype, we next examined whether the defects result from reduced vessel growth or vessel regression. To this end, we normalized vessel density in the mutants against cortical area expansion during the postnatal period (**Fig. 6M**). We found that, in the control brain after accounting for cortical expansion, normalized vessel density significantly increased during the periods both from P3 to P5 (*P* = 9.5×10^−8^, n = 11) and from P5 to P7 (*P* = 2.1×10^−6^, n = 12). By contrast, in mutants, although the normalized vessel density also increased from P3 to P5 (*P* = 10^−6^, n = 9), it grew at a slower rate. Moreover, there were no significant changes from P5 to P7 (*P* = 0.77, n = 12). This indicates that vessel net growth is significantly reduced in mutants from P3 to P7 and basically stops from P5 to P7. Analysis of normalized vessel branch point frequency also showed a similar pattern (**Fig. 6N**). In control brains, normalized branching frequency increased significantly during the period from P3 to P5 (*P* = 0.0003, n = 10), and marginally during the period from P5 to P7 (*P* = 0.019, n = 9). By contrast, in mutants, normalized branching remained not significantly changed during the period either from P3 to P5 (*P* = 0.29, n = 9) or from P5 to P7 (*P* = 0.17, n = 9). This indicates that, unlike in the control brain where significant branching accompanies vessel growth, there is minimal new branch formation in the mutants during these periods. Moreover, analysis of linear branch point density along vessels showed that controls and mutants had a similar profile, with marginal differences at P3 (control, 11±1.2/mm; mutant, 12±1.7 /mm; *P* = 0.78, n = 9), P5 (control, 8±0.6/mm; mutant, 6±0.4/mm; *P* = 0.03, n = 9), or P7 (control, 7±0.3/mm; mutant, 5±0.4/mm; *P* = 0.04, n = 4) (**Fig. 5O**). This indicates that the periodicity of branch formation along vessels is similar between controls and mutants at all stages, suggesting an absence of excessive branch pruning along mutant vessels during the postnatal period. Lastly, to directly assess potential involvement of vessel regression, we performed terminal deoxynucleotidyl transferase dUTP nick end labeling (TUNEL) of endothelial cells. We found that although TUNEL positive cells were observed in the mutant cortex, none of them co-labeled with the endothelial marker IB4 (**Fig. S6**). This indicates that there is no significant cell death along mutant vessels. Thus, these results together indicate that although there are severe defects in vessel development in *orc3* mutants, there is no obvious vessel regression. The vessel defects are therefore likely due to reduced growth of new vessels but not regression of vessels already formed.

**Figure 7:**
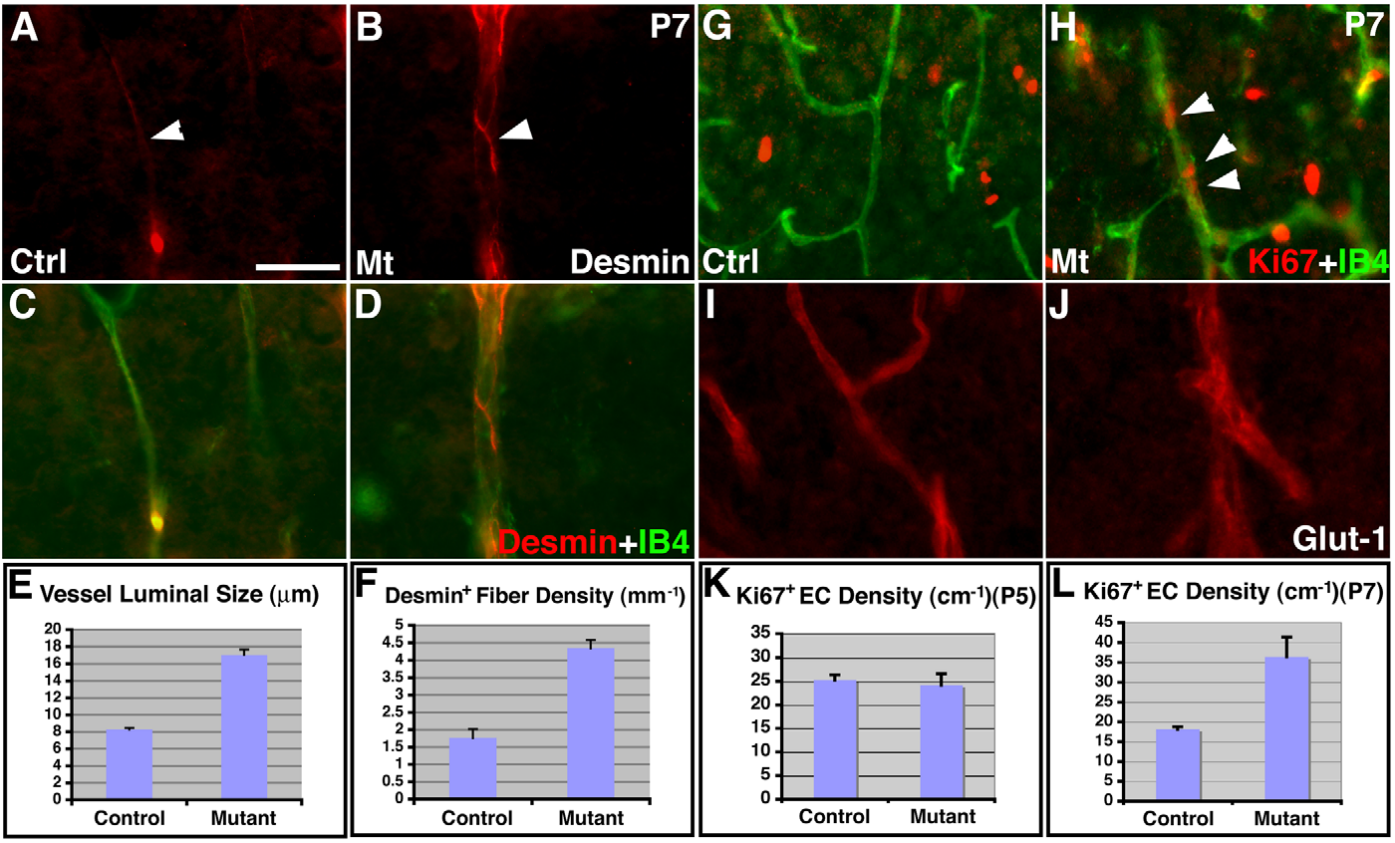
Altered vascular gene expression and endothelial proliferation in class II *orc3/hGFAP-cre* mutant cortices at P7. (**A–D**) Enlarged luminal size as well as increased Desmin (in red) expression along cortical vessels (IB4 staining, in green) in mutants (**A** & **C**) as compared to controls (**B** & **D**) at P7. (**E**) Quantification of vessel luminal size at P7. Average luminal size in mutants was more than doubled (205%) than in controls (control, 8.28±0.22 μm; mutant, 17.05±0.67 μm; *P* = 1.4×10^−13^, n = 25). (**F**) Quantification of Desmin positive pericyte fiber density at P7. The average number of continuous Desmin positive fiber per unit vessel length was more than doubled (247%) in mutants than in controls (control, 1.76±0.22/mm; mutant, 4.35±0.23/mm; *P* = 0.002, n = 3). (**G–H**) Increased Ki67 (in red) positive endothelial cells (IB4 staining, in green) in mutants (**H**) as compared to controls (**G**) at P7. (**I–J**) Similar levels of Glut-1 (in red) expression along cortical vessels between controls (**I**) and mutants (**J**) at P7. (**K–L**) Quantitative analysis shows no significant changes in the density of Ki67 positive endothelial cells along vessels at P5 (control, 25.2±1.1/cm; mutant, 24.2±2.4/cm; *P* = 0.69, n = 12) (**K**), but a 100% increase in mutants at P7 (control, 18.2±0.6/cm; mutant, 36.4±4.9/cm; *P* = 0.002, n = 13) (**L**). Scale bar in (**A**), 50 μm for (**A–J**).

To further determine the effects of astroglial loss on cortical vessel development, we examined mutant endothelial cells and pericytes at P7 (**Fig. 7A–D**). We found that, at P7, mutant vessels appeared enlarged throughout the cortex. Indeed, quantification showed that mutant vessels were on average more than twice as large as control vessels (control, 8.28±0.22 μm; mutant, 17.05±0.67 μm; *P* = 1.4×10^−13^, n = 25) (**Fig. 7E**). Moreover, mutant vessels were frequently surrounded by an increased number of fibers that were strongly positive for Desmin, an intermediate filament protein expressed in pericytes. Quantification showed that the average number of Desmin positive fiber per unit vessel length was more than doubled in mutants than in controls (control, 1.76±0.22/mm; mutant, 4.35±0.23/mm; *P* = 0.002, n = 3) (**Fig. 7F**). These results indicate that with the loss of close interactions with astroglial endfeet, cortical vessels may compensate by increasing vessel luminal size as well as enhancing cytoskeletal gene expression in vascular support cells. To determine mechanisms underlying the increase in vessel luminal size, we next examined endothelial cell proliferation in mutants at both P5 and P7. Interestingly, we found that while no obvious differences were observed between controls and mutants in Ki67 staining along cortical vessels at P5 (data not shown), many more IB4 positive endothelial cells stained positive for Ki67 in mutants at P7 (**Fig. 7G and 7H**). Quantification showed that while the density of Ki67 positive cells along vessels was not significantly different between controls and mutants at P5 (control, 25±1.1/cm; mutant, 24±2.4/cm; *P* = 0.69, n = 12) (**Fig. 7K**), the density was doubled in mutants at P7 (control, 18±0.6/cm; mutant, 36±4.9/cm; *P* = 0.002, n = 13) (**Fig. 7L**). Thus, these results indicate that with loss of close interactions with astroglia, cortical vessels may increase endothelial cell proliferation, potentially contributing to vessel enlargement at P7.

### Recovery of cortical vascular network following astrogliosis

**Figure 8:**
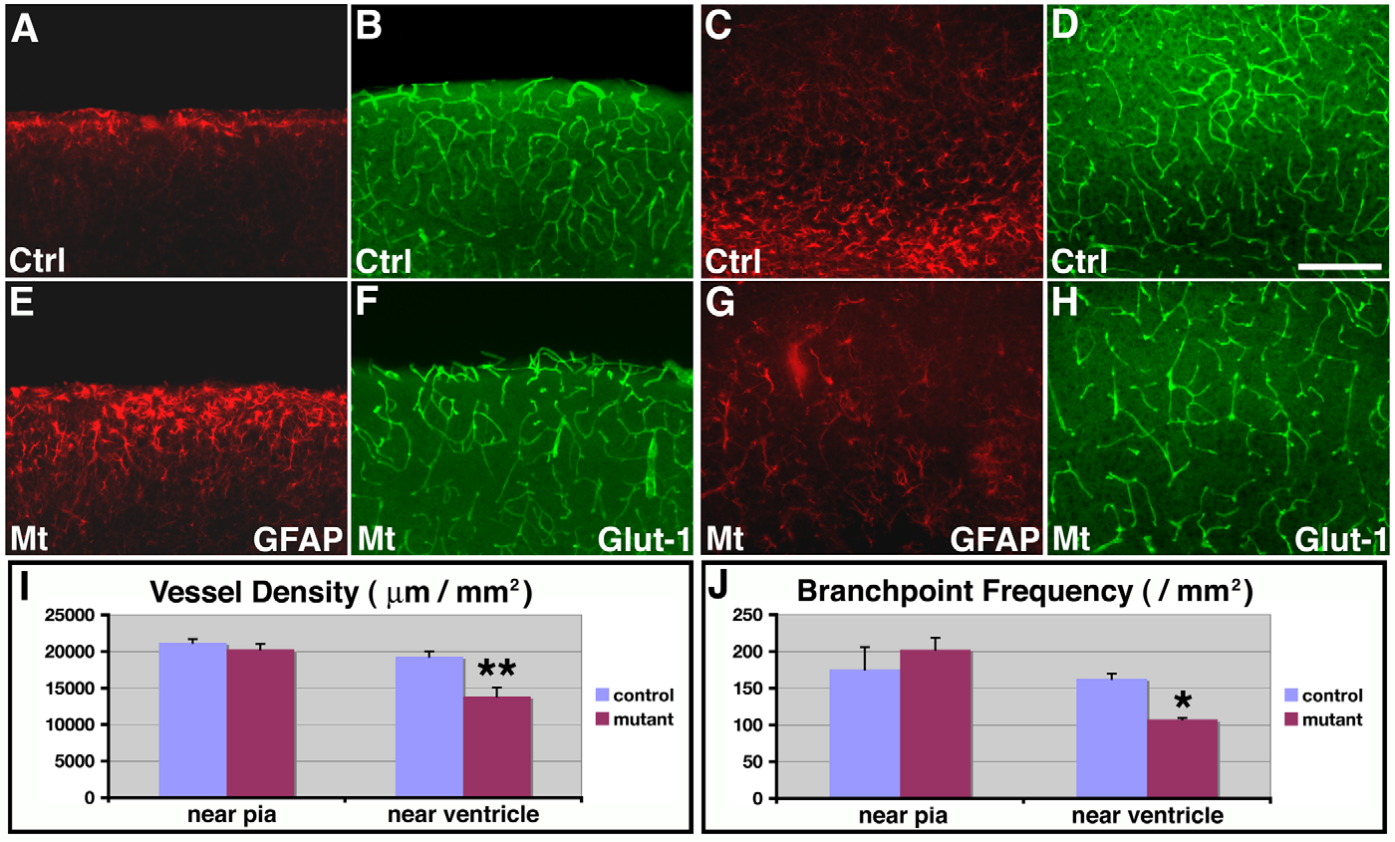
Recovery of vessel development in the upper cortex of class II *orc3/hGFAP-cre* mutants at later postnatal stages. (**A–H**) Astrocytes and glial progenitors were stained using anti-GFAP antibodies (red in **A, C, E & G**) and blood vessel morphology was assessed by anti-Glut-1 staining (green in **B, D, F & H**) in control (**A–D**) and class II mutant (**E–H**) cortices at P14. Dramatic increases in GFAP expression in astrocytes near the pia were observed in mutants as compared to controls (compare **E** to **A**), while the number of glial progenitors near the ventricle remained relatively depleted in mutants (compare **G** to **C**). On the other hand, while vessel density remained low near the ventricle in mutants (compare **H** to **D**), vessel density in the upper cortex has recovered to normal in mutants (compare **E** to **B**). (**I–J**) Quantitative analysis of vessel density (**I**) and branching frequency (**J**) in control and mutants at P14. In cortical regions near the ventricle, significantly lower vessel density (**) (control, 16.7±30.4/mm^2^; mutant, 13837±1216 μm/mm^2^; *P* = 0.008, n = 4) and branching frequency (*) (control, 162.7±7.0/mm^2^; mutant, 107.5±1.9/mm^2^; *P* = 0.011, n = 4) were observed in mutants as compared to controls. By contrast, in regions near the pia, both vessel density (control, 21189±506 μm/mm^2^; mutant, 20271±736 μm/mm^2^; *P* = 0.35, n = 4) and branching frequency (control, 175.7±30.4/mm^2^; mutant, 202.3±16.2/mm^2^; *P* = 0.49, n = 4) in mutants have recovered to a level not significantly different from controls. Scale bar in (**D**), 100 μm for (**A–H**).

Our results above implicate a functional involvement of cortical astrocytes in promoting brain angiogenesis during the early postnatal period. To further determine effects of reduced astrogliogenesis on cortical vessel development, we next examined control and mutant brains at P14. To this end, we used an anti-GFAP antibody to stain for astrocytes and an anti-Glut-1 antibody to stain for blood vessels. We found that in the control cortex at P14, there were two main regions with relatively strong expression of GFAP, one near the pia and the other near the ventricle (**Fig.** **8A and 8C**). In the mutant cortex, we found that, while GFAP staining intensity remained weak in areas near the ventricle (**Fig. 8G**), GFAP expression was dramatically up-regulated in areas near the pia (**Fig. 8E**). This suggests that astrogliosis has taken place near the cortical pia. Interestingly, we found that although GFAP expression was strongly up-regulated in mutants at P14, Ki67 staining in the cortex remained low (data not shown). BrdU labeling also revealed minimal overlap with GFAP positive cells in the mutant cortex (**Fig. S7**). This is consistent with *orc3* gene being deleted from the astroglial lineage by *hGFAP-cre*. It also suggests that astrogliosis in these brains takes place in the absence of astrocyte proliferation. To evaluate cortical vessel morphology, we next examined Glut-1 staining. Interestingly, we found that in the mutant cortex at P14, while vessel development remained defective in areas near the ventricle (compare **Fig. 8H** to **8D**), vessel morphology appeared to have substantially recovered in cortical regions near the pia (compare **Fig. 8F** to **8B**). Indeed, quantitative analysis showed that, in regions near the pia, there were no significant differences between controls and mutants at P14 in either vessel density (control, 21189±506 μm/mm^2^; mutant, 20271±736 μm/mm^2^; *P* = 0.35, n = 4) or branch point frequency (control, 175.7±30.4/mm^2^; mutant, 202.3±16.2/mm^2^; *P* = 0.49, n = 4) (**Fig. 8I–J**). By contrast, in cortical regions near the ventricle where no obvious astrogliosis was observed, significant reductions in vessel density (control, 16.7±30.4 μm/mm^2^; mutant, 13837±1216 μm/mm^2^; *P* = 0.008, n = 4) and branching frequency (control, 162.7±7.0/mm^2^; mutant, 107.5±1.9/mm^2^; *P* = 0.011, n = 4) were still observed in the mutants (**Fig. 8I–J**). Thus, these results indicate that following an early delay in vessel development, enhanced astrocytic function from astrogliosis appears to be sufficient for substantially restoring vessel growth at a later stage. This provides further support for our interpretation that cortical astrocytes play a functionally critical role in promoting brain angiogenesis during early postnatal development.

## Discussion

It is long known that endothelial cells closely interact with astrocytes during blood vessel development in the retina [6]. Genetic studies have also recently implicated significant requirement for the function of several genes in atrocytes during retinal angiogenesis [8], [9]. These findings suggest a role of astrocytes in regulating vessel development throughout the central nervous system. In this article, we show that astrocytes play a functionally critical role in promoting vascular network elaboration in the early postnatal cerebral cortex. We show that astrocytes interact closely with blood vessels in the early postnatal brain (**Fig. 1**). We also find that deletion of *orc3* gene using *hGFAP-cre* severely inhibits cortical astrogliogenesis (**Fig. 2** and **3**), but largely spares neurogenesis or oligogenesis (**Fig. 4**). This in turn leads to significant reductions in astrocytic process coverage of the cortex and decreased blood vessel sprouting (**Fig. 5**). As a result, both vessel density and branching frequency are reduced during early postnatal corticogenesis, a phenotype that we show is associated with reduced vessel growth but not regression (**Fig. 6**). Furthermore, we find that loss of astrocytic interaction results in apparently compensatory changes in vascular cells, including increased vessel luminal size as well as enhanced cytoskeletal protein expression in pericytes (**Fig. 7**). Lastly, we show that the reduced vessel density and branching are substantially rescued following astrogliosis at later stages (**Fig. 8**). Thus, these results not only demonstrate a novel approach for determining cell type function through lineage specific cell cycle blockade, but also uncover a functionally critical role of astrocytes in promoting brain vessel development. This not only suggests a potentially universal role of astrocytes in promoting angiogenesis throughout the central nervous system, but may also facilitate better understanding of a large number of brain diseases that involves perturbed neurovascular interactions.

### Inhibition of astrogliogenesis by *orc3* deletion from cortical neural progenitors

Neural progenitors are long known to give birth first to neurons and then to glia during brain development [15]. By taking advantage of the *hGFAP-cre*, a *cre* line known to first become active during mid to late embryonic corticogenesis, together with a conditional allele of an *orc3* gene essential for cell cycle progression, we show that *orc3* deletion by *hGFAP-cre* results in a relatively specific inhibition of cortical astrogliogenesis. Indeed, we find that early deletion of *orc3* results in severe loss of astrocytes, leading to cortical degeneration. Later deletion of *orc3*, on the other hand, results in reduced astrocyte production, but largely preserves cortical neurogenesis and neuronal differentiation. This provides a unique opportunity to determine the functional involvement of astrocytes in regulating brain vessel development. Indeed, our studies provide the first functional evidence that implicates a role of astroglia in promoting corticical vessel growth and branching during development. In principle, given a lineage specific *cre* line for targeting relevant progenitor cells, this approach may be adapted for determining the cellular function of any cell type(s). Thus, our conditional *orc3* allele will likely be a useful genetic tool for elucidating cell type specific function both inside and outside the nervous system.

### Roles of astrocytes in normal blood vessel development in the brain

In the retina, vessel development is closely associated with astrocytic gene expression. A number of genes, including R-cadherin, fibronectin, heparan sulfate glycosyltransferase, vascular endothelial growth factor (VEGF), and αvβ8 integrin, have been implicated in this process by various approaches [8], [9], [10], [26] (but also see [11], [12]). We have found that, in the cortex, inhibition of astrogliogenesis also results in reduced vessel growth and branching during the postnatal period. This indicates a similar role of astrocytes in promoting blood vessel development in the brain. Although the underlying molecular mechanisms remain to be determined, our results suggest a potentially direct role of astrocytes in promoting brain angiogenesis. First, similar to earlier studies of the rat cortex [17], we find an intimate interaction between astrocytic processes and blood vessels in the postnatal mouse brain. This indicates a direct interaction between astrocytes and blood vessels during brain development. Second, we find that, while late deletion of *orc3* by *hGFAP-cre* results in dramatically reduced astrogliogenesis, it largely spares cortical neurogenesis and neuronal differentiation. Indeed, we not only observe normal expression of several laminar specific neuronal markers, but also observe normal neurofilament expression as well as minimal neuronal cell death. Furthermore, we observe, in the mutant cortex, relatively normal levels of all splice isoforms of VEGF A (data not shown), a pro-angiogenic factor expressed near exclusively in cortical neurons during the early postnatal period [27]. Thus, although we cannot exclude the possibility of altered neuronal expression of other genes involved in brain angiogenesis, it appears likely that astrocytes in the brain play a direct role in regulating vessel development. Lastly, we find that enhanced astrocytic function from gliosis at later stage appears to substantially rescue cortical vessel development, which provides further support for the interpretation of a specific role by astrocytes. Thus, in analogy to the retina, our results strongly suggest a direct role of astrocytes in promoting vascular network elaboration during postnatal brain development. Such interpretation is also consistent with recent studies showing that astrocytes are responsible for orchestrating the development of a parallel blood vessel scaffold in the rostral migratory stream [28].

### Roles of astrocytes in brain vascular disease and recovery

Astrocytes form a near complete covering of the vasculature through their perivascular endfeet in the adult brain, and are known to be a key mediator of neurovascular coupling. Indeed, it is increasingly recognized that dys-regulated astrocytic signaling and function play a critical role in the pathophysiology of a large number of cerebral vascular diseases [22], [23]. In cerebral ischemia, for example, increased production of reactive oxygen species and mitochondrial dysfunction in hippocampal CA1 astrocytes has been found to contribute to ischemia-induced loss of glutamate transporter Glt-1 and ultimately the death of CA1 neurons [29]. Our results show that reduced astrogliogenesis in the postnatal cerebral cortex can also result in defective vessel network development. This suggests that, besides in the adult brain, defective astrogliogensis or perturbed astrocyte function during development may also play a role in the pathology of cerebrovascular diseases and may be of relevance to better understanding these diseases. On the other hand, angiogenesis in peri-infarct regions has been observed in rodent models of cerebral ischemia as well as in human stroke, and has been suggested to potentially play a role in facilitating recovery after stroke [30], [31]. Our implication of astrocyte function in promoting normal postnatal brain angiogenesis during development and of astrogliosis in restoring vessel morphology following early developmental defects suggests that enhancing the pro-angiogenic function of astrocytes may also be a potentially beneficial strategy for improved recovery after stroke.

## Materials and Methods

### Mouse care and breeding

This study was approved by the Animal Care and Use Committee of the University of Wisconsin-Madison (Animal Welfare Assurance # A3368-01). *nestin-creER* (Stock #: 012906) *and mTomato/mEGFP* reporter (Stock #: 007576) lines were purchased from the Jackson Laboratory. Tamoxifen (3 mg per animal) was administered intraperitoneally at E18.0 for *nestin-creER* induced *mTomato/mEGFP* reporter recombination. *hGFAP-cre* line was kindly provided by Dr. Albee Messing (UW-Madison) (Zhuo et al., 2001). *hGFAP-cre* was introduced into the *orc3* conditional mutant background for phenotypic analyses and *orc3* homozygotes without *cre* and heterozygotes with *cre* were both analyzed as controls. Noon after vaginal plug is regarded as E0.5, and day of birth as P0. For S phase labeling, 5-bromo-deoxyuridine (BrdU) was injected at 100 μg/gbw and embryos were dissected 4 hours later for fixation and processing.

### Immunohistochemistry

The following primary antibodies were used at respective dilutions/concentrations: mouse anti-BrdU supernatant (clone G3G4, Developmental Studies Hybridoma Bank (DSHB), University of Iowa, IA; 1∶40), mouse anti-vimentin supernatant (clone 40E–C, DSHB; 1∶20), mouse anti-neurofilament supernatant (clone 2H3, DSHB; 1∶20), rabbit anti-BLBP (Millipore; 1∶500), rabbit anti-Cux1 (CDP) (Santa Cruz; 1∶100), rat anti-Ctip2 (Abcam, 1∶500), rabbit anti-Laminin (Sigma; 1∶2000), rabbit anti-Collagen IV (AbD Serotec; 1∶500), rabbit anti-Glut-1 (Thermo Scientific; 1∶200), rabbit anti-GFAP (Dako;1∶500), rat anti-CD140a (PDGFRα) (Pharmingen; 1∶125), rabbit anti-Ki67 Ab-4 (Thermo Scientific; 1∶100), rabbit anti-Desmin (Millipore; 1∶500), mouse anti-CD31 (PECAM-1) supernatant (clone 2H8, DSHB; 1∶10), rat anti-CD31 (BD Pharmingen; 1∶100), and biotinylated isolectin B4 (Vector Lab; 40 μg/ml). FITC and Cy3 conjugated secondary antibodies were purchased from Jackson ImmunoResearch Laboratories (West Grove, PA). Peroxidase conjugated secondary antibodies were purchased from Santa Cruz Biotech. Staining procedures were performed as described previously [32], and sections were mounted with Fluoromount G medium (Southern Biotech, Birmingham, AB) and analyzed under a Nikon *eclipse* Ti microscope or an Olympus confocal microscope. For 3-D reconstruction, NIH ImageJ software was employed. TUNEL staining was performed using an in situ cell death detection kit (Roche) per manufacture's instructions.

### Quantitative analysis

Every sixth of 50 μm thick coronal sections from each brain, representing the entire anterior-posterior axis, were stained with IB4, and vessel length and branching frequency were quantified manually using the NIS-Elements BR 3.0 software from Nikon. Similarly, for astrocyte numbers, oligodendrocyte precursor numbers, and Ki67 positive cell numbers, matching regions from control and mutant samples were used and cell numbers were manually counted. For vessel sprout quantification, to exclude sectioning artifacts, only terminal protrusions with smooth endings were included. Astrocytic process coverage was measured using ImageJ software after applying same threshold parameters for all conditions. Statistical analyses were performed using Student's *t* test when two conditions are involved, and using ANOVA followed by Tukey's *post hoc* test when more than two conditions are involved. All data are represented as mean ± SEM.

## Supporting Information

Figure S1 **Schematic of** ***orc3*** **gene targeting approach.** The *orc3* genomic locus in embryonic stem (ES) cells was targeted using an engineered BAC vector that contained an *orc3* genomic fragment where exons 5–7 were flanked by a pair of loxP sites. A *pgk-neomycin* selection cassette was in addition inserted near the 5′ lox P site. After germline transmission, the *neomycin* selection cassette was removed using an *actin-FLPe* transgenic line to derive the *orc3* conditional allele.
(TIF)

Figure S2 **Gross morphology of class I and class II** ***orc3/hGFAP-cre*** **mutants at P3, P7 and P14.** Severe hemorrhage was observed in class I mutants at P3. Cortical degeneration (arrows) was observed in class I mutants at P7 and P14 (note that because of long storage in buffer, hemorrhage was no longer obvious in P7 brains). No apparent hemorrhage or degeneration was observed in class II mutants at any stage, although these brains were also smaller than controls of the same stage.
(TIF)

Figure S3 **Differential effects of** ***orc3*** **deletion on subventricular zone cell proliferation in class I and class II mutants.** Ki67 staining (in red) revealed a large number of proliferating cells in the subventricular zone in control animals at P3 (**A**). The number was severely reduced in class I mutants (**B**) but only moderately affected in class II mutants (**C**) at this stage. ANOVA analysis of Ki67 positive cells per field showed significant differences between all three comparisons (**D**), between controls (115.8±4.7), class I mutants (46.3±4.3), and class II mutants (73.1±7.2). Scale bar in (**C**), 100 μm for (**A–C**).
(TIF)

Figure S4 **Severe loss of cortical astrocytes in class I** ***orc3/hGFAP-cre*** **mutants at P0 and P3 and its effects on vessel development.** (**A–D**) Control and mutant brain sections were stained for the young astrocyte marker BLBP (in red). Severe reductions in the number of BLBP positive cells were observed in mutants (**B**&**D**), as compared to controls (**A**&**C**), at both P0 (**A**&**B**) and P3 (**C**&**D**). (**E–F'**) Vessel morphology in control and class I mutant cortices at P3. IB4 staining (in green) revealed a severe underdevelopment of blood vessels in the mutant cortex. (**G–H**) Quantification of vessel density and branching frequency in control and class I mutant cortices at P3. Significant reductions in vessel density were observed in class I (control, 15109±1139 μm/mm^2^; mutant 6868±516 μm/mm^2^; *P*<0.001; n = 7), but not class II (control, 15109±1139 μm/mm^2^; mutant 13027±753 μm/mm^2^; *P* = 0.25; n = 9) mutants at P3. Similarly, significant reductions in branching frequency were observed in class I (control, 160.9±10.2/mm^2^; mutant 22.4±2.6/mm^2^; *P*<0.001; n = 7), but not class II (control, 160.9±10.2/mm^2^; mutant 146.0±14.6/mm^2^; *P* = 0.59; n = 9) mutants at P3. Statistical analysis was performed using one-way ANOVA followed by Tukey's post hoc test. Scale bar in (**A**), 200 μm for (**A–F'**).
(TIF)

Figure S5 **Analysis of apoptosis in class II** ***orc3/hGFAP-cre*** **mutant cortices at P7.** Staining against active caspase 3 showed occasional positive cells (arrows) in both control (**A** & **A'**) and class II *orc3/hGFAP-cre* mutant (**B** & **B'**) cortices at P7. Sccale bar in (**A**), 100 μm for all panels.
(TIF)

Figure S6 **TUNEL and IB4 staining in class II** ***orc3/hGFAP-cre*** **mutant cortices at P7.** Although we observed occasional TUNEL positive (in green) cells in the mutant cortex (arrowhead in **B**), no co-localizing with the endothelial marker IB4 (in red) was observed. This indicates that the lower vessel density in class II mutants is not a result of endothelial cell death. Sccale bar in (**A**), 100 μm for both panels.
(TIF)

Figure S7 **Astrogliosis in P14 class II mutants is not accompanied by astrocyte proliferation.** BrdU staining (in green) revealed occasionally positive cells in both control (**A**) and class II mutant (**B**) cortices at P14. Despite strongly up-regulated GFAP (in red) expression, however, astrocytes near the pia in mutant cortices were not positive for BrdU staining. This is consistent with *orc3* gene deletion from the astrocytes. Sccale bar in (**A**), 100 μm for both panels.
(TIF)

Movie S1 **Three-dimensional reconstruction of interactions between processes of an astrocyte and a blood vessel in the wildtype mouse cerebral cortex at P7.**
(MP4)

Movie S2 **Three-dimensional reconstruction of interactions between a second astrocyte and a blood vessel in the wildtype mouse cerebral cortex at P7.**
(MP4)
