# Supplementary figures and images for: A Functional Requirement for Astroglia in Promoting Blood Vessel Development in the Early Postnatal Brain
Source: PLoS One. 2012 Oct 24;7(10):e48001. doi: 10.1371/journal.pone.0048001 (PMC3480497; doi:10.1371/journal.pone.0048001)

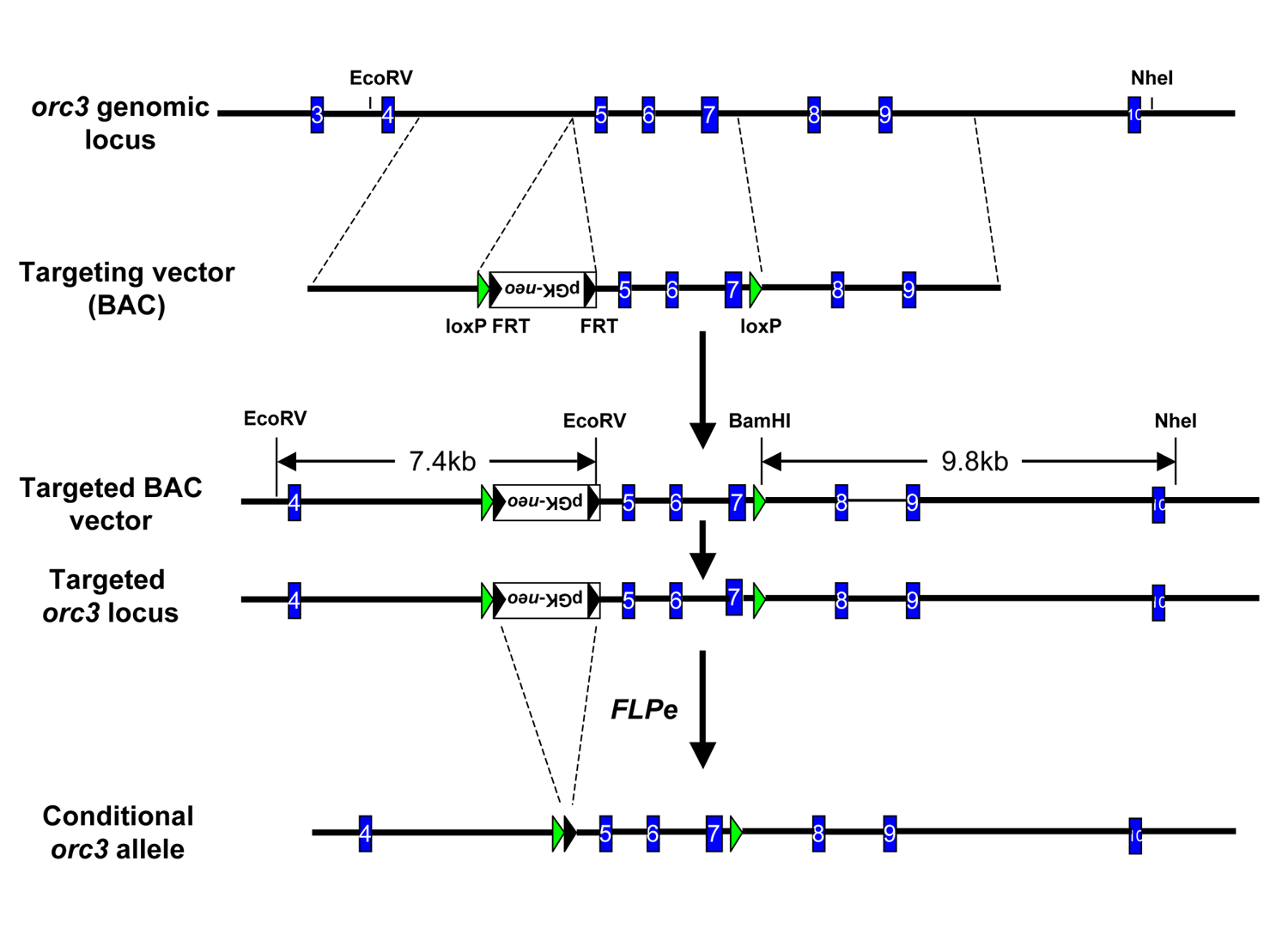

Supplement: Figure S1 — Schematic of orc3 gene targeting approach. The orc3 genomic locus in embryonic stem (ES) cells was targeted using an engineered BAC vector that contained an orc3 genomic fragment where exons 5–7 were flanked by a pair of loxP sites. A pgk-neomycin selection cassette was in addition inserted near the 5′ lox P site. After germline transmission, the neomycin selection cassette was removed using an actin-FLPe transgenic line to derive the orc3 conditional allele. (TIF) [file pone.0048001.s001.tif]

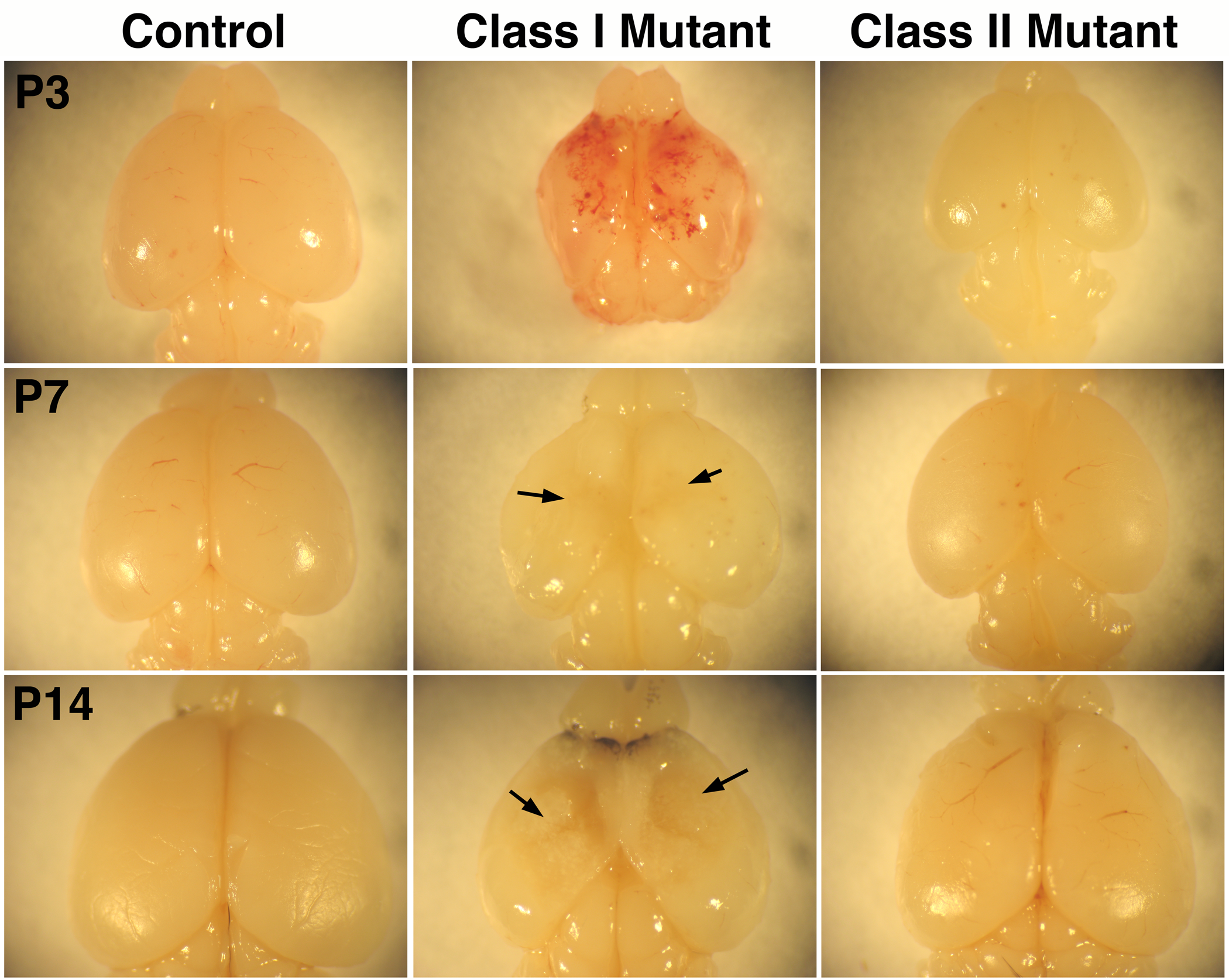

Supplement: Figure S2 — Gross morphology of class I and class II orc3/hGFAP-cre mutants at P3, P7 and P14. Severe hemorrhage was observed in class I mutants at P3. Cortical degeneration (arrows) was observed in class I mutants at P7 and P14 (note that because of long storage in buffer, hemorrhage was no longer obvious in P7 brains). No apparent hemorrhage or degeneration was observed in class II mutants at any stage, although these brains were also smaller than controls of the same stage. (TIF) [file pone.0048001.s002.tif]

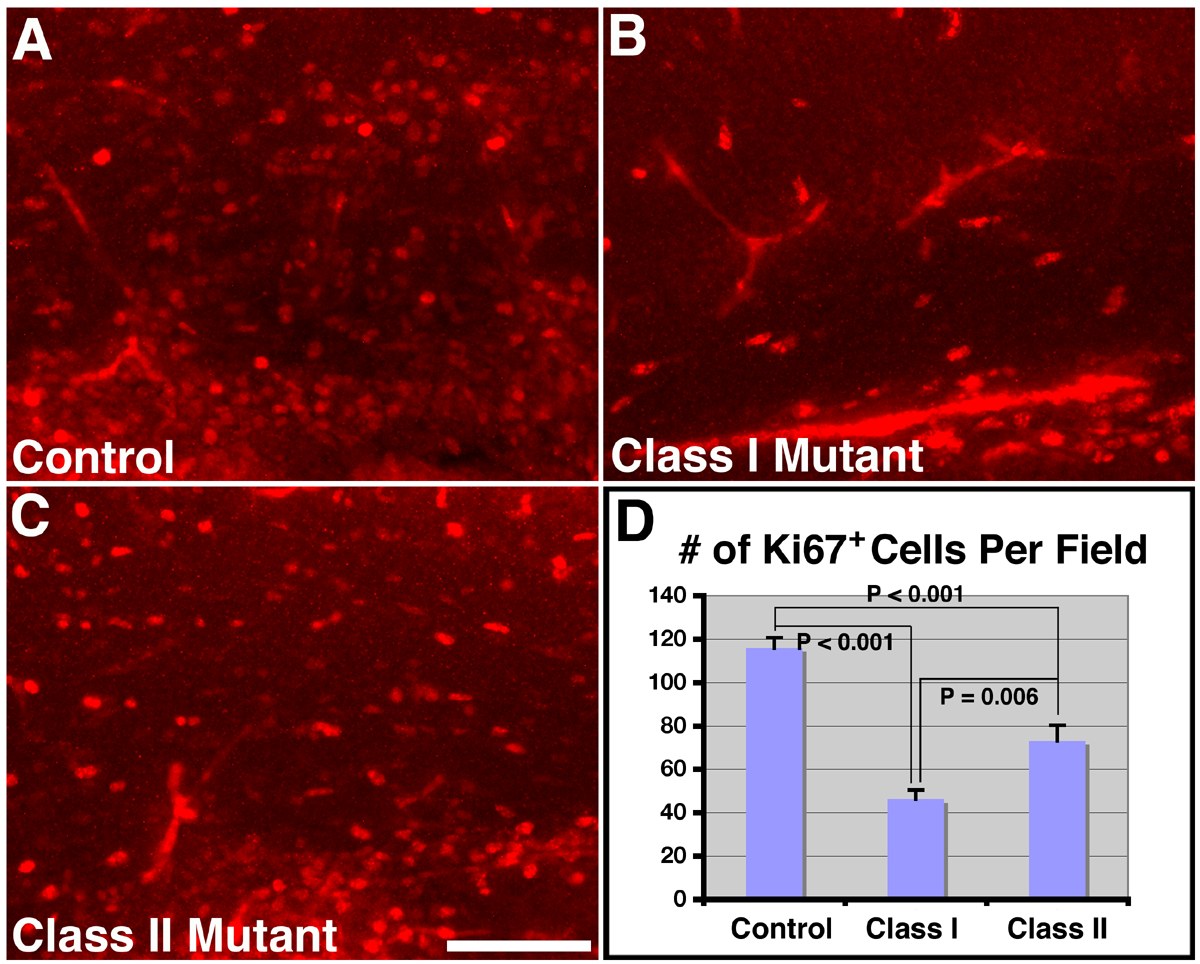

Supplement: Figure S3 — Differential effects of orc3 deletion on subventricular zone cell proliferation in class I and class II mutants. Ki67 staining (in red) revealed a large number of proliferating cells in the subventricular zone in control animals at P3 (A). The number was severely reduced in class I mutants (B) but only moderately affected in class II mutants (C) at this stage. ANOVA analysis of Ki67 positive cells per field showed significant differences between all three comparisons (D), between controls (115.8±4.7), class I mutants (46.3±4.3), and class II mutants (73.1±7.2). Scale bar in (C), 100 μm for (A–C). (TIF) [file pone.0048001.s003.tif]

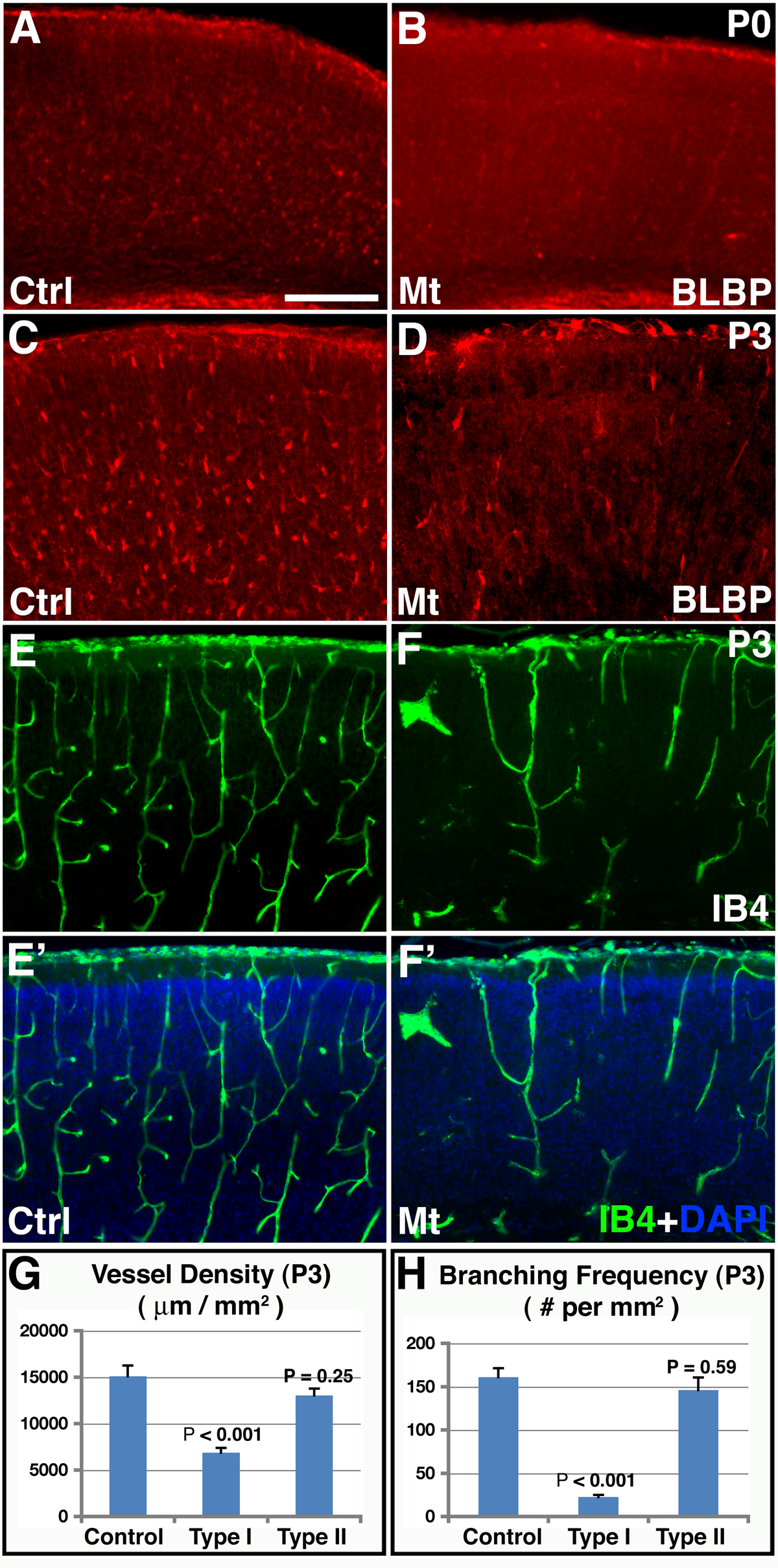

Supplement: Figure S4 — Severe loss of cortical astrocytes in class I orc3/hGFAP-cre mutants at P0 and P3 and its effects on vessel development. (A–D) Control and mutant brain sections were stained for the young astrocyte marker BLBP (in red). Severe reductions in the number of BLBP positive cells were observed in mutants (B&D), as compared to controls (A&C), at both P0 (A&B) and P3 (C&D). (E–F') Vessel morphology in control and class I mutant cortices at P3. IB4 staining (in green) revealed a severe underdevelopment of blood vessels in the mutant cortex. (G–H) Quantification of vessel density and branching frequency in control and class I mutant cortices at P3. Significant reductions in vessel density were observed in class I (control, 15109±1139 μm/mm2; mutant 6868±516 μm/mm2; P<0.001; n = 7), but not class II (control, 15109±1139 μm/mm2; mutant 13027±753 μm/mm2; P = 0.25; n = 9) mutants at P3. Similarly, significant reductions in branching frequency were observed in class I (control, 160.9±10.2/mm2; mutant 22.4±2.6/mm2; P<0.001; n = 7), but not class II (control, 160.9±10.2/mm2; mutant 146.0±14.6/mm2; P = 0.59; n = 9) mutants at P3. Statistical analysis was performed using one-way ANOVA followed by Tukey's post hoc test. Scale bar in (A), 200 μm for (A–F'). (TIF) [file pone.0048001.s004.tif]

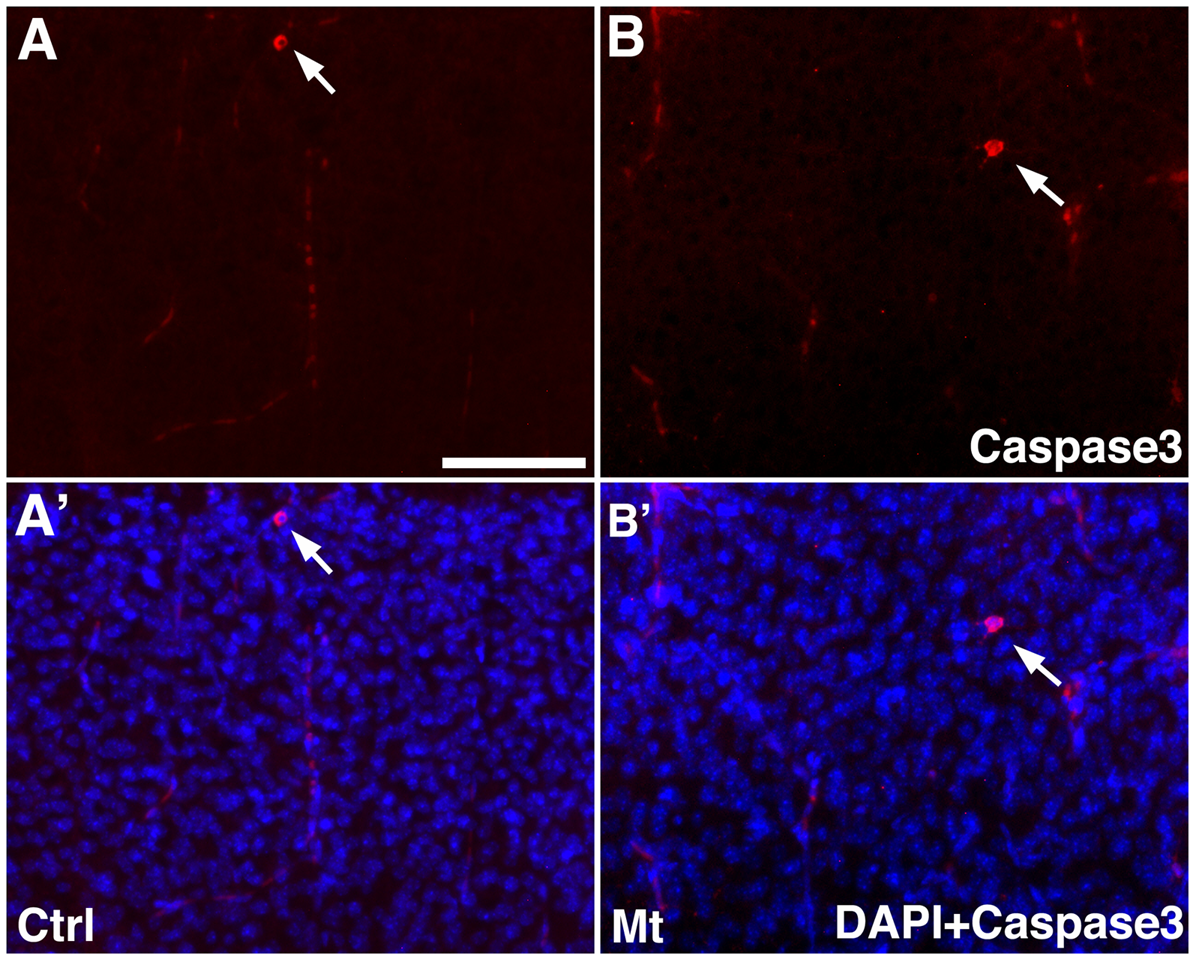

Supplement: Figure S5 — Analysis of apoptosis in class II orc3/hGFAP-cre mutant cortices at P7. Staining against active caspase 3 showed occasional positive cells (arrows) in both control (A & A') and class II orc3/hGFAP-cre mutant (B & B') cortices at P7. Sccale bar in (A), 100 μm for all panels. (TIF) [file pone.0048001.s005.tif]

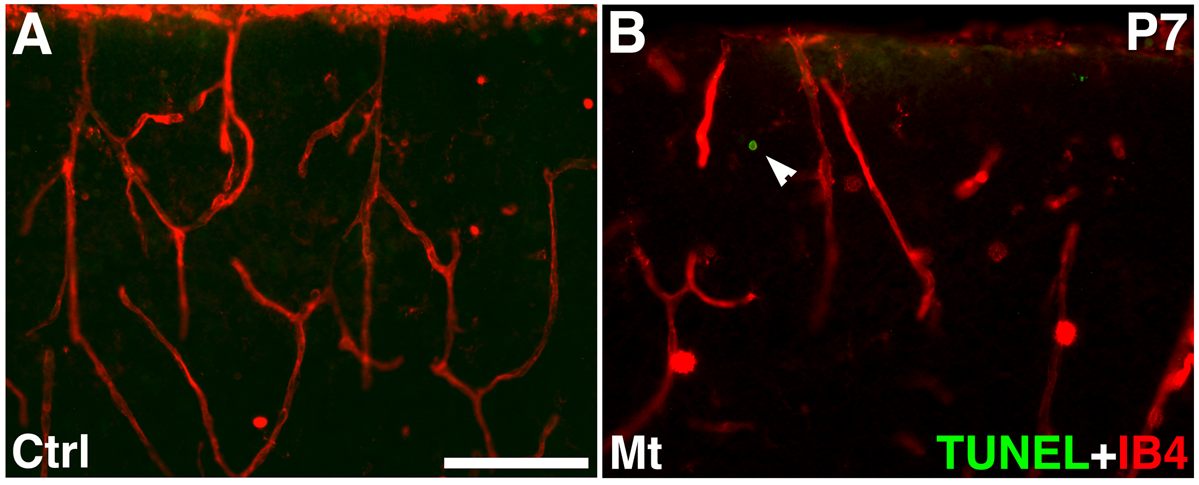

Supplement: Figure S6 — TUNEL and IB4 staining in class II orc3/hGFAP-cre mutant cortices at P7. Although we observed occasional TUNEL positive (in green) cells in the mutant cortex (arrowhead in B), no co-localizing with the endothelial marker IB4 (in red) was observed. This indicates that the lower vessel density in class II mutants is not a result of endothelial cell death. Sccale bar in (A), 100 μm for both panels. (TIF) [file pone.0048001.s006.tif]

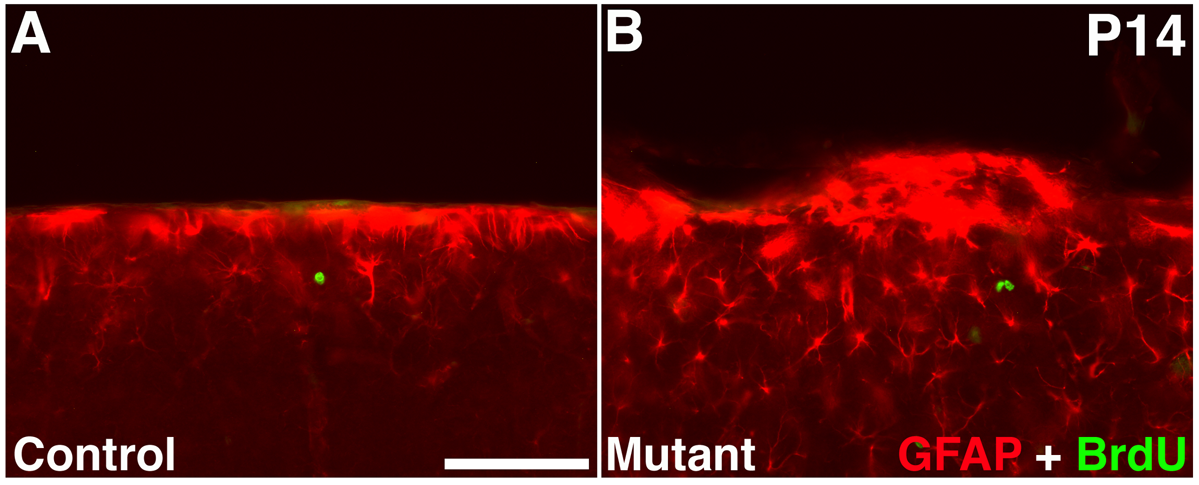

Supplement: Figure S7 — Astrogliosis in P14 class II mutants is not accompanied by astrocyte proliferation. BrdU staining (in green) revealed occasionally positive cells in both control (A) and class II mutant (B) cortices at P14. Despite strongly up-regulated GFAP (in red) expression, however, astrocytes near the pia in mutant cortices were not positive for BrdU staining. This is consistent with orc3 gene deletion from the astrocytes. Sccale bar in (A), 100 μm for both panels. (TIF) [file pone.0048001.s007.tif]
